# Supplementary material for: Activation of Ca2+ transport in cardiac microsomes enriches functional sets of ER and SR proteins
Source: Mol Cell Biochem. 2023 Apr 10;479(1):85–98. doi: 10.1007/s11010-023-04708-0 (PMC10786961; doi:10.1007/s11010-023-04708-0)
Supplement: Supplementary file 1 — Supplementary file1 (DOCX 252 KB) [file 11010_2023_4708_MOESM1_ESM.docx]

**Appendix**


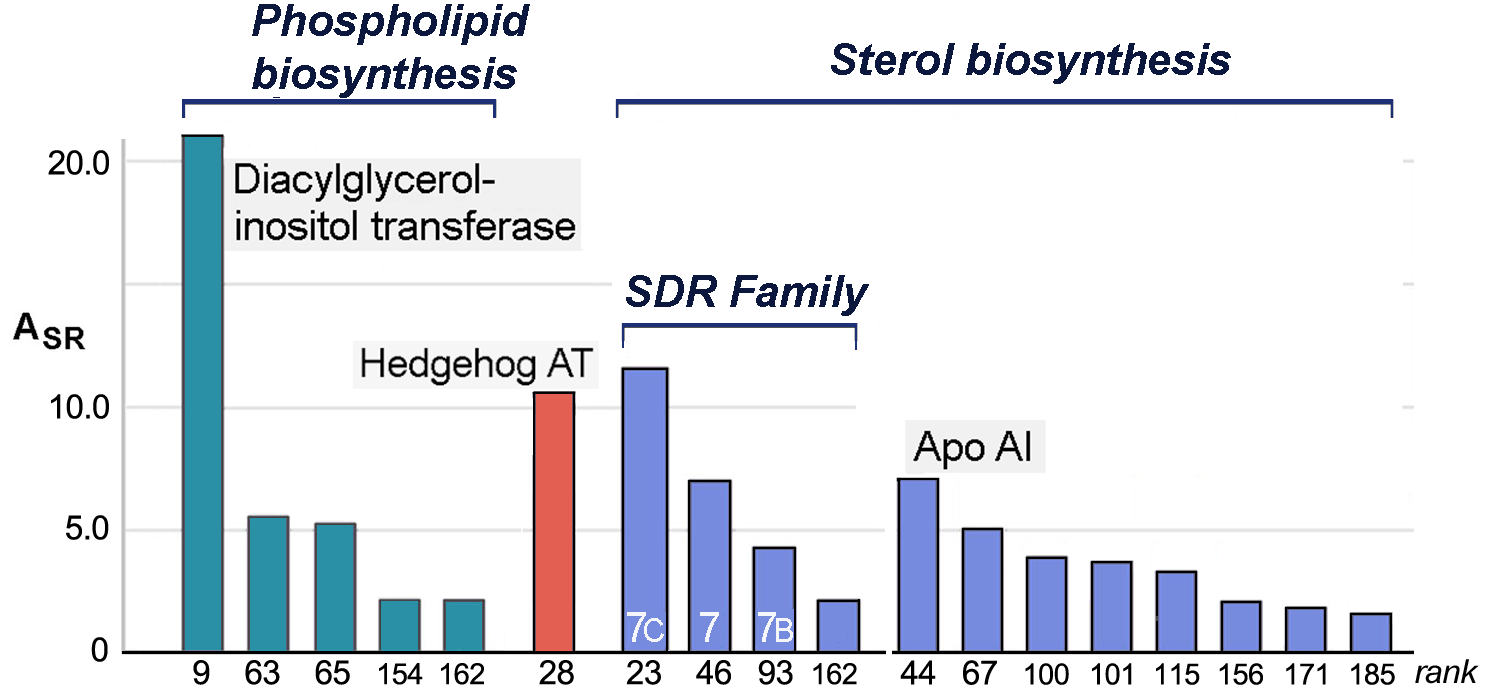


**Fig. 1.**  **Lipid metabolism proteins enriched in SERCA-positive SR.** Relative spectral abundances (A_SR_, SERCA=100.0) for groups of enzymes expected to function in phospholipid and cholesterol biosynthesis. The highest A_SR_-value lipid biosynthetic enzyme was CDP-diacylglycerol-inositol 3-phosphatidyltransferase (A_SR_ = 20.7). Other acyltransferase (AT) enzymes are involved with formation of phospholipids from diacylglycerides. Hedgehog acyltransferase-like (A_SR_ = 10.5) may act to palmitoylate rab proteins. Short-chain dehydrogenase/ reductase (SDR) enzymes are prominent oxidoreductase enzymes; isotypes are indicated in white. Various proteins were present that are active in cholesterol and/or sterol biosynthesis (e.g., Apo-A1). Rank identifies proteins by their order among 354 SERCA-positive SR proteins sorted by A_SR_ (see Online Resource 1).

| Protein | Rank | A_SR_ | E_SR_ | E_sub_ |
| --- | --- | --- | --- | --- |
| Histone H4 | 8 | 21. | 3.6 | 0.38 |
| Histone H2A | 60 | 5.7 | 4.8 | 0.37 |
| Histone H2B | 110one 110 | 3.4 | 2.2 | 0.57 |
| Histone macro-H2A.1 | 208 | 1.3 | 4.0 | 0.89 |
| Histone H3.2 | 210 | 1.3 | 5.4 | 0.62 |
| *Mean ± S.D.* |  |  | 4.0±1.2 | 0.57±0.21 |

**Table 1. Histones enriched in SERCA-positive SR membranes.** Histone H4 peptides were detected at a level (A_SR_) greater than the other histone proteins combined. All histones were enriched an average of 4.0-fold over crude cardiac microsomes (E_SR_), and were, on the average, 57% more enriched in the denser microsomes (E_sub_ = +0.57), which entails the more rigorous requirement for SERCA activity. Rank is the order among 354 proteins identified in SERCA-positive SR sorted by A_SR_ (see Online Resource 1).

| **Protein** | **MW** | **Rank** | **A_SR_** | **E_SR_** | **E_sub_** |
| --- | --- | --- | --- | --- | --- |
| Desmin | 53 | 2 | 95.8 | 9.5 | 0.27 |
| Alpha Crystallin B-chain | 20 | 12 | 18.3 | 5.0 | 0.07 |
| Vimentin 12 | 54 | 18 | 13.8 | 8.4 | 0.37 |
| Keratin II 6B | 61 | 47 | 6.9 | 2.8 | 0.25 |
| β-tubulin 2C | 50 | 48 | 6.8 | 4.0 | -0.04 |
| Synemin | 141 | 106 | 3.7 | 43. | 0.40 |

**Table 2. Filamentous proteins enriched with SERCA-positive SR membranes.** A_SR_ levels are normalized to that of SERCA2a (=100), and Rank is the order among 354 proteins identified in SERCA-positive SR, sorted by A_SR_ (see Online Resource 1). Cytoskeleton proteins (monomers) varied greatly in E_SR_ and E_sub_, likely because they do not enrich with particular ER/SR functional domains, unlike proteins that are in the membranes.

|  | - Protein | - MW | - A_SR_ | - E_SR_ | - E_sub_ |
| --- | --- | --- | --- | --- | --- |
| - 1 | - Malectin | - 32 | - 7.8 | 8.5 | - 0.14 |
| - 2 | - Dol-diPO_4_--protein glycosyltransferase subunit DAD1 | - 12 | - 6.1 | - 10. | - 0.39 |
| - 3 | - signal peptidase complex subunit 2 | - 25 | - 5.8 | - 9.7 | - 0.16 |
| - 4 | - translocon-associated protein subunit delta | - 19 | - 5.7 | - 4.0 | - -0.08 |
| - 5 | - mannose-P-dolichol utilization defect 1 protein | - 27 | - 5.0 | - 3.5 | - 0.12 |
| - 6 | - Dol-diPO_4_-protein glycosyltransferase | - 69 | - 3.6 | - 6.0 | - 0.49 |
| - 7 | - Dol-diPO_4_-protein glycosyltransferase subunit 2 | - 69 | - 3.2 | - 5.1 | - 0.13 |
| - 8 | - translocon-associated protein subunit alpha | - 32 | - 3.0 | - 7.0 | - 0.27 |
| - 9 | - Dol-diPO_4_-protein glycosyltransferase 48 kDa subunit | - 50 | - 2.9 | - 3.5 | - 0.37 |
|  | - *Average of 9 most abundant (±S.D.)* |  | - 4.4 ± 1.4 | - 6.1 ±2.7 | - 0.23 ± 0.18 |
| 10 | - UDP-glucose:glycoprotein glucosyltransferase 1 | - 222 | - 1.1 | - 4.8 | - 0.25 |
| - 11 | - protein transport protein Sec61 subunit alpha | - 61 | - 0.65 | - 12. | - 0.39 |
| - 12 | - signal recognition particle receptor subunit alpha | - 70 | - 0.63 | - 5.2 | - 0.35 |
| - 13 | - glucosidase 2 subunit beta | - 60 | - 0.57 | - 8.0 | - 0.38 |
| - 14 | - Dol-diPO_4_-protein glycosyltransferase subunit STT3B | - 94 | - 0.52 | - 23. | - 0.18 |
| - 15 | - mannosyl-oligosaccharide glucosidase | - 92 | - 0.32 | - 13. | - -0.19 |

**Table 3. Rough ER proteins enriched in SERCA-positive SR membranes.** Rough ER proteins play roles in nascent protein translocation (rows 3, 4, 8, 11, 12; 40% of total), while roles in N-linked glycosylation comprised the majority of the proteins, including many involved in formation and transfer of the core oligosaccharide from dolichol (Dol).

| *Membrane transport* | *Rank* | *A_SR_* | *E_SR_* | *E_sub_* |
| --- | --- | --- | --- | --- |
| Ca-binding protein p22 | 25 | 11.9 | 9.3 | 0.17 |
| Sec22b | 27 | 10.5 | 6.0 | 0.41 |
| VAMP-2 | 87 | 4.4 | 2.3 | 0.09 |
| Sec20 | 96 | 4.1 | 26. | 0.49 |
| *Protein traffic* | *Rank* | *A_SR_* | *E_SR_R* | *E_sub_* |
| TMED10 | 35 | 9.0 | 3.5 | -.042 |
| TMED9 | 38 | 8.1 | 9.3 | 0.12 |
| TMED2 | 49 | 6.6 | 8.1 | 0.092 |
| TMED1 | 143 | 2.5 | 6.0 | 0.16 |
| *ER structure/form* | *Rank* | *A_SR_* | *E_SR_* | *E_sub_* |
| reticulon-2 | 53 | 6.2 | 6.7 | 0.44 |
| lunapark-3 | 95 | 4.1 | 6.4 | 0.49 |
| reticulon-4 | 99 | 3.9 | 5.6 | 0.35 |
| climp-63 | 124 | 2.9 | 8.6 | 0.37 |
| atlastin-2 | 266 | 0.71 | 16.5 | 0.48 |

**Table 4. SERCA-positive ER/SR proteins involved in membrane dynamics.** Major protein/lipid/membrane trafficking proteins enriched in SERCA-positive SR. Functionally related sets of proteins involved in protein and/or lipid transport are listed in order of spectral abundance (A_SR_). The average enrichment of these proteins in SERCA-positive SR (E_SR_) compared to crude cardiac microsomes was 7.2 ± 2.0-fold. Rank is the order among 354 SERCA-positive proteins sorted by A_SR_ (see Online Resource 1).
