# Supplementary material for: Activation of Ca2+ transport in cardiac microsomes enriches functional sets of ER and SR proteins
Source: Mol Cell Biochem. 2023 Apr 10;479(1):85–98. doi: 10.1007/s11010-023-04708-0 (PMC10786961; doi:10.1007/s11010-023-04708-0)
Supplement: Supplementary file 2 — Supplementary file2 (PDF 399 KB) Online Resource 1: GeLC-MSMS Data Set used in this study. [file 11010_2023_4708_MOESM2_ESM.pdf]

Table S1. The 354 *bona fide* (SERCA-positive) SR proteins, and 1102 proteins identified in canine cardiac membranes. Proteins discovered for three membrane preparations, MV, MedSR, and HighSR are listed in order of greatest spectral abundance values (A_{SR}), calculated as the spectral levels in MedSR plus HighSR membranes, divided by molecular weight (MW), and normalized to the level of SERCA2 (A_{SR}=100.0). The first 354 proteins have E_{SR} values >2.00; i.e., twice the level of peptide spectra in SR fractions compared to crude microsomes (MVs), and are defined as SERCA-positive SR in this study. The next 496 proteins are, essentially, contaminants in the SR preparation, in that they are less abundant in SR than in the MV preparation (E_{SR} values < 2.00). The final 253 proteins are mostly low abundance proteins for which enrichment in SR fractions could not be determined because none was detected in the cruder starting material. The enrichment factor E_{SR} is the spectral abundance of each protein in MedSR and HighSR compared to levels in crude MVs. Colored cells (*leftmost column*) correspond to the color key shown in Fig. 9.

no.	see Fig 9	Identified Proteins (1103)	MW	Total Spectra			ASR	ESR	Esub
				MV	MedSR	HighSR			
354 proteins enriched in SR (ESR values ≥2.0). Proteins listed in order of decreasing relative ASR									
1	Ca	SERCA2	110.0	1144.5	1503.8	2445.0	100.0	3.5	0.24
2		desmin	53.0	192.3	667.2	1156.0	95.8	9.5	0.27
3	Lum	sarcalumenin	54.0	306.8	413.5	1006.2	73.2	4.6	0.42
4	Ca	phospholamban	6.0	17.7	41.1	68.6	50.9	6.2	0.25
5	Lum	calsequestrin-2	47.0	147.9	397.6	295.4	41.1	4.7	-0.15
6		NADH-cytochrome b5 reductase 3	34.0	100.3	151.0	209.3	29.5	3.6	0.16
7	rCyc	PRA1 family protein 3	22.0	38.7	68.9	128.3	25.0	5.1	0.30
8		histone H4-like	11.0	23.2	25.8	57.2	21.0	3.6	0.38
9	PL	CDP-diacylglycerol--inositol 3-phosphatidyltransferase isoform 1	23.0	29.9	58.9	112.1	20.7	5.7	0.31
10	teth	vesicle-associated membrane protein-associated protein B	27.0	32.0	53.7	125.8	18.5	5.6	0.40
11		fat storage-inducing transmembrane protein 2	30.0	40.6	70.6	128.7	18.5	4.9	0.29
12		alpha-crystallin B chain isoform 5	20.0	26.0	61.4	70.2	18.3	5.1	0.07
13	KDEL	78 kDa glucose-regulated protein isoform 5	72.0	88.8	112.9	334.1	17.3	5.0	0.49
14	jSR	aspartyl/asparaginyl beta-hydroxylase	23.0	21.5	80.9	56.8	16.7	6.4	-0.17
15	Ca	sarcoplasmic/endoplasmic reticulum calcium ATPase 1 isoform 11	110.0	82.0	233.6	371.0	15.3	7.4	0.23
16		transmembrane protein 109	26.0	21.7	69.1	73.7	15.3	6.6	0.03
17		membrane-associated progesterone receptor component 2	30.0	19.1	60.1	89.5	13.9	7.8	0.20
18		vimentin isoform 12	54.0	31.4	83.1	180.0	13.6	8.4	0.37
19	Perox	malate dehydrogenase, cytoplasmic isoform 1	36.0	26.6	125.6	43.4	13.1	6.4	-0.49
20		peptidyl-prolyl cis-trans isomerase B isoform 2	24.0	22.8	49.1	57.2	12.3	4.7	0.08
21	Perox	peroxisomal multifunctional enzyme type 2 isoform 2	80.0	22.4	253.2	92.3	12.0	15.4	-0.47
22		ankyrin-1-like	18.0	13.2	36.4	40.2	11.8	5.8	0.05
23		dehydrogenase/reductase SDR family member 7C isoform 1	35.0	15.4	58.6	85.6	11.5	9.3	0.19
24	teth	vesicle-associated membrane protein-associated protein A isoform 2	28.0	15.4	40.2	73.6	11.3	7.4	0.29
25	mTr	calcium-binding protein p22 isoform 1	22.0	9.5	36.5	51.9	11.2	9.3	0.17
26	rCyc	prenylcysteine oxidase	57.0	32.8	83.1	142.2	11.0	6.9	0.26
27	mTr	vesicle-trafficking protein SEC22b	25.0	15.6	28.0	66.2	10.5	6.0	0.41
28	rCyc	hedgehog acyltransferase-like isoform 1	57.0	42.1	119.9	94.6	10.5	5.1	-0.12
29		sarcolemmal membrane-associated protein isoform 2	93.0	70.1	114.1	215.9	9.9	4.7	0.31
30	Perox	L-lactate dehydrogenase B chain	37.0	19.4	99.5	30.6	9.8	6.7	-0.53
31		delta(3,5)-Delta(2,4)-dienoyl-CoA isomerase, mitochondrial isoform 1	36.0	11.1	97.4	29.0	9.8	11.4	-0.54
32	KDEL	protein disulfide-isomerase A3	57.0	31.8	62.4	133.4	9.6	6.2	0.36
33	Perox	catalase	60.0	3.9	163.6	40.7	9.5	53.0	-0.60
34		catechol O-methyltransferase	25.0	9.3	22.5	59.6	9.1	8.8	0.45
35	p24	transmembrane emp24 domain-containing protein 10 isoform 1	25.0	22.8	42.0	38.6	9.0	3.5	-0.04
36	KDEL	endoplasmin precursor	93.0	49.2	65.1	217.2	8.5	5.7	0.54
37		redox-regulatory protein PAMM	25.0	10.1	30.6	43.5	8.3	7.3	0.17
38	p24	transmembrane emp24 domain-containing protein 9 isoform 1	27.0	8.5	34.4	44.2	8.1	9.3	0.12
39		glycerol-3-phosphate dehydrogenase 1-like	38.0	12.0	77.4	29.3	7.8	8.9	-0.45
40	glyc	malectin isoform 2	32.0	10.5	38.5	50.8	7.8	8.5	0.14
41		calnexin precursor	68.0	27.8	60.7	128.1	7.7	6.8	0.36
42	rab	ras-related protein Rab-2A	24.0	31.8	34.6	30.0	7.5	2.0	-0.07
43		thioredoxin-related transmembrane protein 2 isoform 1	34.0	9.5	32.2	57.4	7.3	9.5	0.28
44		apolipoprotein A-I	30.0	28.8	45.1	31.8	7.1	2.7	-0.17
45		alpha-actinin-2 isoform 3	104.0	62.3	119.3	139.6	6.9	4.2	0.08
46		dehydrogenase/reductase SDR family member 7	38.0	20.2	38.6	55.7	6.9	4.7	0.18
47		keratin, type II cytoskeletal 6B	61.0	53.8	56.7	93.6	6.9	2.8	0.25
48		tubulin beta-2C chain isoform 1	50.0	30.2	63.1	58.5	6.8	4.0	-0.04
49	p24	transmembrane emp24 domain-containing protein 2 isoform 1	23.0	6.8	24.6	29.6	6.6	8.0	0.09
50		PQ-loop repeat-containing protein 3 isoform 1	23.0	12.3	24.1	28.8	6.4	4.3	0.09
51		BAG family molecular chaperone regulator 2	24.0	4.2	15.5	39.1	6.3	13.0	0.43
52		CDGSH iron-sulfur domain-containing protein 2-like	15.0	3.9	18.4	15.4	6.3	8.8	-0.09
53	struc	reticulon-2 isoform 2	51.0	16.9	31.8	81.0	6.2	6.7	0.44
54	glyc	dolichyl-diphosphooligosaccharide--protein glycosyltransferase subunit DA	12.0	2.6	8.0	18.2	6.1	10.2	0.39
55	teth	junctophilin-2	46.0	31.9	60.2	37.9	5.9	3.1	-0.23

56	glyc	signal peptidase complex subunit 2	25.0	5.3	21.7	30.3	5.8	9.7	0.16
57	glyc	translocon-associated protein subunit delta precursor	19.0	9.8	20.9	17.9	5.7	4.0	-0.08
58	rab	ras-related protein Rab-1A	23.0	13.5	25.3	21.6	5.7	3.5	-0.08
59		histone H2A type 2-C-like	14.0	5.9	8.9	19.5	5.7	4.8	0.37
60		polymerase I and transcript release factor-like	43.0	40.0	52.9	32.7	5.5	2.1	-0.24
61		basigin	29.0	22.7	29.2	27.5	5.4	2.5	-0.03
62	jSR	ryanodine receptor 2	565.0	441.7	731.2	364.1	5.4	2.5	-0.34
63	PL	long-chain fatty acid transport protein 1 isoform 1	71.0	24.9	57.1	80.4	5.4	5.5	0.17
64	rab	ras-related protein Rab-1B isoform 1	22.0	13.5	20.5	21.6	5.3	3.1	0.03
65	PL	lysophospholipid acyltransferase 5 isoform 1	56.0	14.9	33.7	72.1	5.3	7.1	0.36
66		vacuolar ATPase assembly integral membrane protein VMA21	11.0	2.6	8.0	12.6	5.2	8.0	0.23
67		neutral cholesterol ester hydrolase 1	46.0	20.1	32.0	52.4	5.1	4.2	0.24
68		peroxiredoxin-1	22.0	18.9	20.2	20.1	5.1	2.1	0.00
69		cytochrome b5	15.0	5.1	10.6	16.8	5.1	5.3	0.23
70	glyc	mannose-P-dolichol utilization defect 1 protein	27.0	13.9	21.3	27.3	5.0	3.5	0.12
71		heat shock protein beta-1	23.0	6.3	15.1	25.9	5.0	6.5	0.26
72		delta-sarcoglycan	32.0	15.7	35.2	21.8	5.0	3.6	-0.24
73	KDEL	protein disulfide-isomerase A2	58.0	19.8	27.5	73.6	4.9	5.1	0.46
74		trimeric intracellular cation channel type A	33.0	4.4	21.3	35.9	4.8	12.9	0.25
75		transmembrane protein 111-like isoform 1	30.0	10.7	13.1	38.5	4.8	4.8	0.49
76		glutathione S-transferase kappa 1 isoform 2	26.0	8.5	34.1	10.4	4.8	5.2	-0.53
77		keratin, type II cytoskeletal 75	59.0	24.3	34.5	66.2	4.8	4.1	0.32
78		membrane-associated progesterone receptor component 1	22.0	2.7	17.5	19.8	4.7	13.8	0.06
79		peroxisomal bifunctional enzyme	80.0	2.3	105.1	29.8	4.7	59.3	-0.56
80	rab	ras-related protein Rab-6A	24.0	8.1	24.0	16.5	4.7	5.0	-0.19
81		isocitrate dehydrogenase [NAD] subunit alpha, mitochondrial isoform 1	40.0	26.2	49.6	17.4	4.7	2.6	-0.48
82		peroxiredoxin-4-like isoform 1	29.0	7.6	9.9	38.3	4.6	6.3	0.59
83		retinol dehydrogenase 14-like	37.0	7.0	26.2	34.2	4.5	8.6	0.13
84	rab	ras-related protein Rab-7a	24.0	12.2	23.8	14.4	4.4	3.1	-0.25
85		caveolin-1	21.0	13.6	22.7	10.7	4.4	2.5	-0.36
86		glycophorin-A precursor	14.0	1.2	21.0	1.2	4.4	18.7	-0.89
87	mTr	vesicle-associated membrane protein 2	13.0	9.0	9.3	11.2	4.4	2.3	0.09
88		transmembrane and coiled-coil domain-containing protein 1	21.0	4.0	13.3	19.3	4.3	8.2	0.18
89		acyl-CoA dehydrogenase family member 11-like	87.0	5.3	107.3	27.8	4.3	25.4	-0.59
90		transmembrane protein 33	28.0	2.7	13.2	30.3	4.3	16.0	0.39
91	KDEL	protein disulfide-isomerase A4 isoform 3	72.0	14.3	33.4	78.0	4.3	7.8	0.40
92		peptidyl-prolyl cis-trans isomerase FKBP1B isoform 2	12.0	3.9	9.8	8.4	4.2	4.7	-0.08
93		dehydrogenase/reductase SDR family member 7B	35.0	3.5	22.6	30.1	4.2	14.9	0.14
94	KDEL	protein disulfide-isomerase A6	48.0	4.7	14.5	57.1	4.2	15.1	0.59
95	struc	protein lunapark isoform 3	48.0	11.2	20.3	51.1	4.1	6.4	0.43
96	mTr	vesicle transport protein SEC20 isoform 1	26.0	1.5	9.8	28.7	4.1	26.1	0.49
97		alpha-2-macroglobulin receptor-associated protein	42.0	5.8	30.1	31.4	4.1	10.7	0.02
98	rab	ras-related protein Rab-5C	23.0	8.9	18.1	14.6	4.0	3.7	-0.11
99	struc	reticulon-4 isoform 1	131.0	33.1	60.1	125.4	3.9	5.6	0.35
100		estradiol 17-beta-dehydrogenase 12 isoform 2	34.0	8.3	24.9	23.2	3.9	5.8	-0.04
101		hydroxysteroid dehydrogenase-like protein 2	46.0	8.6	42.4	21.2	3.8	7.4	-0.33
102		probable glutathione peroxidase 8	24.0	2.0	21.7	11.5	3.8	16.9	-0.31
103		plectin isoform 7	517.0	51.2	222.9	484.4	3.8	13.8	0.37
104		myozenin-2	30.0	4.8	17.0	23.5	3.8	8.4	0.16
105		endoplasmic reticulum resident protein 29	40.0	9.6	23.3	29.6	3.7	5.5	0.12
106		synemin	141.0	4.3	55.7	129.6	3.7	42.8	0.40
107	glyc	dolichyl-diphosphooligosaccharide--protein glycosyltransferase subunit 1 is	69.0	14.9	22.8	66.2	3.6	6.0	0.49
108		probable saccharopine dehydrogenase	41.0	8.6	15.7	36.3	3.5	6.0	0.40
109	rab	ras-related protein Rab-11B	24.0	12.2	17.8	12.3	3.5	2.5	-0.18
110		histone H2B type 2-E-like	14.0	7.9	3.8	13.5	3.4	2.2	0.57
111		platelet-activating factor acetylhydrolase precursor	50.0	14.0	22.2	39.3	3.4	4.4	0.28
112	rab	ras-related protein Rab-14 isoform 1	24.0	12.4	15.5	13.4	3.4	2.3	-0.07
113		heme oxygenase 2 isoform 2	36.0	3.7	15.3	27.8	3.3	11.5	0.29
114		vesicular integral-membrane protein VIP36 precursor	40.0	1.2	19.3	28.3	3.3	40.9	0.19
115		apolipoprotein E isoform 1	37.0	8.7	22.1	21.5	3.3	5.0	-0.02

116	glyc	dolichyl-diphosphooligosaccharide--protein glycosyltransferase subunit 2 is	69.0	15.4	34.4	44.7	3.2	5.1	0.13
117		serpin H1 precursor	47.0	4.4	15.0	37.6	3.1	11.8	0.43
118	glyc	translocon-associated protein subunit alpha precursor	32.0	5.0	12.8	22.1	3.0	7.0	0.27
119		heat shock protein beta-6	17.0	6.0	9.7	8.7	3.0	3.1	-0.06
120		alkyldihydroxyacetonephosphate synthase, peroxisomal	73.0	2.3	62.0	16.8	3.0	34.7	-0.57
121		dephospho-CoA kinase domain-containing protein	26.0	8.1	16.9	10.8	3.0	3.4	-0.22
122		isochorismatase domain-containing protein 1	33.0	2.4	27.5	7.5	3.0	14.8	-0.57
123		protein-L-isoaspartate(D-aspartate) O-methyltransferase isoform 5	30.0	7.4	12.9	18.8	2.9	4.3	0.18
124	struc	cytoskeleton-associated protein 4 (CLIMP-63)	65.0	7.8	21.2	46.5	2.9	8.6	0.37
125	glyc	dolichyl-diphosphooligosaccharide--protein glycosyltransferase 48 kDa sub	50.0	14.7	16.2	35.5	2.9	3.5	0.37
126		ATP-binding cassette sub-family D member 3	68.0	5.0	52.1	18.3	2.9	13.9	-0.48
127		ATP-binding cassette sub-family D member 1	90.0	6.1	70.8	20.1	2.8	15.0	-0.56
128		LDLR chaperone MESD	29.0	1.5	8.3	20.5	2.8	19.5	0.42
129		tetratricopeptide repeat protein 35-like	35.0	3.5	15.2	19.4	2.8	9.9	0.12
130		flotillin-1 isoform 2	47.0	17.8	24.4	22.1	2.8	2.6	-0.05
131		gamma-sarcoglycan	32.0	3.6	15.0	16.4	2.7	8.8	0.05
132		isocitrate dehydrogenase [NAD] subunit beta, mitochondrial isoform 1	42.0	13.3	23.0	18.0	2.7	3.1	-0.12
133		trans-2,3-enoyl-CoA reductase-like isoform 2	42.0	2.5	26.2	14.4	2.7	16.3	-0.29
134		L-lactate dehydrogenase A chain isoform 1	37.0	4.8	25.7	9.9	2.7	7.4	-0.44
135		neighbor of COX4	24.0	1.4	11.0	12.0	2.7	17.0	0.04
136		NADPH--cytochrome P450 reductase	76.0	14.7	29.5	43.2	2.7	5.0	0.19
137		ras-related protein R-Ras	24.0	10.4	15.0	7.8	2.6	2.2	-0.32
138		peroxisomal acyl-coenzyme A oxidase 2-like	77.0	7.1	48.5	23.3	2.6	10.1	-0.35
139		tubulin alpha-1B chain	51.0	10.0	25.4	21.3	2.6	4.7	-0.09
140		3-hydroxyacyl-CoA dehydrogenase type-2 isoform 1	27.0	5.9	14.0	10.2	2.5	4.1	-0.16
141		UPF0480 protein C15orf24 homolog isoform 1	26.0	2.0	11.3	11.8	2.5	11.4	0.02
142	KDEL	calreticulin isoform 4	48.0	5.0	9.6	32.4	2.4	8.5	0.54
143		hydroxyacyl-coenzyme A dehydrogenase, mitochondrial	36.0	15.2	22.2	9.0	2.4	2.0	-0.42
144		transmembrane emp24 domain-containing protein 1	25.0	3.5	8.9	12.2	2.4	6.0	0.16
145	KDEL	protein disulfide-isomerase	57.0	6.7	14.1	33.3	2.3	7.0	0.41
146		acetolactate synthase-like protein-like	68.0	9.8	28.1	27.6	2.3	5.7	-0.01
147	rab	ras-related protein Rab-18 isoform 2	26.0	9.5	12.5	8.7	2.3	2.2	-0.18
148		testis-expressed sequence 264 protein isoform 2	34.0	3.5	7.7	19.8	2.3	7.9	0.44
149		myeloid-associated differentiation marker-like	35.0	8.1	17.1	11.2	2.3	3.5	-0.21
150		vinculin	127.0	32.9	53.8	48.6	2.2	3.1	-0.05
151		CAAX prenyl protease 1 homolog	54.0	5.2	13.3	28.9	2.2	8.2	0.37
152		10 kDa heat shock protein, mitochondrial isoform 1	13.0	3.9	7.2	2.8	2.1	2.6	-0.44
153		protease, serine, 3 isoform 3	26.0	6.7	11.0	9.0	2.1	3.0	-0.10
154		lysocardiolipin acyltransferase 1	45.0	2.5	11.4	22.9	2.1	13.8	0.34
155		keratin, type I cytoskeletal 17 isoform 1	48.0	9.9	11.0	25.3	2.1	3.7	0.39
156		17-beta-hydroxysteroid dehydrogenase type 6 isoform 3	36.0	1.2	8.4	18.8	2.1	22.9	0.38
157		protein canopy homolog 4	28.0	1.2	5.3	15.8	2.1	17.8	0.50
158	rab	ras-related protein Rab-5B isoform 1	24.0	7.4	10.9	7.2	2.1	2.5	-0.21
159	KDEL	protein disulfide-isomerase TMX3	51.0	5.7	13.2	25.2	2.1	6.8	0.31
160	JSR	triadin	78.0	9.5	38.5	19.9	2.1	6.2	-0.32
161		fatty aldehyde dehydrogenase	54.0	8.6	17.0	23.2	2.1	4.7	0.16
162		1-acyl-sn-glycerol-3-phosphate acyltransferase beta isoform 2	31.0	6.5	11.7	11.3	2.1	3.6	-0.02
163		dehydrogenase/reductase SDR family member 4	27.0	1.6	17.1	2.9	2.1	12.5	-0.71
164		platelet glycoprotein 4	53.0	16.3	24.7	14.1	2.0	2.4	-0.27
165		selenoprotein S	21.0	2.0	3.8	11.5	2.0	7.7	0.50
166		protein RER1	23.0	2.0	6.7	9.7	2.0	8.1	0.18
167		3-hydroxyacyl-CoA dehydratase 2	37.0	5.1	15.3	10.6	1.9	5.1	-0.18
168		hydroxymethylglutaryl-CoA lyase, mitochondrial-like isoform 1	34.0	6.7	22.5	1.2	1.9	3.5	-0.90
169		ankyrin repeat and SOCS box protein 11 isoform 1	35.0	1.2	9.2	14.9	1.9	20.3	0.24
170		DnaJ (Hsp40) homolog, subfamily B, member 11	42.0	1.2	7.5	20.6	1.9	24.2	0.46
171		epoxide hydrolase 1 isoform 2	52.0	7.8	11.2	23.3	1.8	4.4	0.35
172		caveolin-2	18.0	2.0	9.5	2.3	1.8	5.8	-0.61
173		thy-1 membrane glycoprotein isoform 2	18.0	4.4	8.8	2.9	1.8	2.7	-0.51
174		flotillin-2-like	53.0	16.7	14.8	19.6	1.8	2.1	0.14
175		peptidyl-prolyl cis-trans isomerase FKBP2 isoform 2	15.0	1.3	4.0	5.6	1.8	7.5	0.17

176	beta-sarcoglycan	35.0	3.1	9.8	12.6	1.8	7.2	0.13
177	nicalin	63.0	5.8	13.3	26.9	1.8	7.0	0.34
178	60S ribosomal protein L23	15.0	3.9	5.3	4.2	1.8	2.5	-0.12
179	LIM domain-binding protein 3 isoform 5	39.0	6.3	9.8	14.8	1.8	3.9	0.21
180	protein LYRIC	64.0	1.3	17.3	22.8	1.7	31.2	0.14
181	band 3 anion transport protein	104.0	10.8	56.5	8.5	1.7	6.0	-0.74
182	V-type proton ATPase 16 kDa proteolipid subunit isoform 1	16.0	2.6	5.5	4.2	1.7	3.8	-0.14
183	calcium/calmodulin-dependent protein kinase type II subunit delta isoform	55.0	4.3	14.3	17.9	1.6	7.5	0.11
184	GPI transamidase component PIG-S	61.0	5.0	13.9	21.5	1.6	7.1	0.21
185	epoxide hydrolase 2 isoform 3	62.0	1.0	27.8	7.6	1.6	36.7	-0.57
186	spectrin alpha chain, brain	285.0	32.1	75.9	86.4	1.6	5.1	0.06
187	calcium signal-modulating cyclophilin ligand	33.0	2.4	6.4	12.3	1.6	7.7	0.32
188	integrin beta-1	88.0	19.0	25.6	23.1	1.5	2.6	-0.05
189	succinyl-CoA ligase [ADP/GDP-forming] subunit alpha, mitochondrial	35.0	9.3	18.2	1.1	1.5	2.1	-0.89
190	filamin-C isoform 4	290.0	34.4	63.3	96.7	1.5	4.7	0.21
191	erlin-2 isoform 4	38.0	4.0	7.9	13.0	1.5	5.2	0.24
192	alpha-soluble NSF attachment protein isoform 1	33.0	7.0	9.0	9.1	1.5	2.6	0.00
193	myosin light polypeptide 6 isoform 1	17.0	1.3	9.3	0.0	1.5	7.2	-1.00
194	acyl-coenzyme A thioesterase 6-like isoform 2	46.0	1.1	19.1	5.3	1.5	22.0	-0.57
195	RING finger protein 170	30.0	2.0	8.7	7.2	1.5	7.8	-0.09
196	lectin, mannose-binding 2-like isoform 4	40.0	1.2	9.1	12.1	1.5	18.2	0.14
197	uncharacterized protein C10orf35	13.0	1.3	2.7	4.2	1.5	5.3	0.23
198	alpha-methylacyl-CoA racemase	42.0	1.9	13.4	8.6	1.5	11.5	-0.22
199	tubulin beta-2B chain isoform 2	50.0	12.4	12.4	13.8	1.5	2.1	0.05
200	ras-related protein Rap-1b isoform 2	21.0	1.5	7.9	2.9	1.4	7.3	-0.47
201	3-hydroxyisobutyrate dehydrogenase, mitochondrial isoform 1	35.0	7.1	16.8	1.2	1.4	2.5	-0.87
202	erythrocyte band 7 integral membrane protein	31.0	7.1	11.2	4.7	1.4	2.2	-0.41
203	sorbin and SH3 domain-containing protein 1 isoform 1	95.0	2.5	28.2	20.1	1.4	19.4	-0.17
204	UBX domain-containing protein 4 isoform 1	57.0	3.0	11.4	17.1	1.4	9.4	0.20
205	alpha-actinin-1 isoform 1	105.0	8.3	25.3	26.7	1.4	6.3	0.03
206	transmembrane protein 43 isoform 1	45.0	1.0	6.6	15.6	1.4	23.0	0.41
207	zinc-binding alcohol dehydrogenase domain-containing protein 2	40.0	1.2	16.3	3.1	1.4	16.7	-0.68
208	core histone macro-H2A.1 isoform 2	40.0	4.6	1.1	17.7	1.3	4.0	0.89
209	spectrin beta chain, brain 1 isoform 1	274.0	22.5	57.3	70.9	1.3	5.7	0.11
210	histone H3.2-like	15.0	1.3	1.3	5.6	1.3	5.4	0.62
211	regulator of microtubule dynamics protein 3	52.0	1.0	8.0	15.7	1.3	24.6	0.33
212	ras-related protein Ral-A isoform 1	24.0	4.4	3.7	7.2	1.3	2.5	0.32
213	nodal modulator 2	134.0	18.9	24.3	35.9	1.3	3.2	0.19
214	cathepsin K precursor	37.0	1.2	12.9	3.3	1.2	14.0	-0.59
215	F-actin-capping protein subunit alpha-1 isoform 2	33.0	2.4	10.9	3.3	1.2	5.9	-0.54
216	uncharacterized protein KIAA0090	112.0	12.5	18.8	28.8	1.2	3.8	0.21
217	sarcoplasmic reticulum histidine-rich calcium-binding protein	83.0	7.0	14.1	20.6	1.2	5.0	0.19
218	uncharacterized protein LOC612691	34.0	1.3	7.2	7.0	1.2	11.0	-0.01
219	popeye domain-containing protein 2	34.0	3.6	6.5	7.7	1.2	4.0	0.09
220	abhydrolase domain-containing protein 16A isoform 1	63.0	3.9	8.4	17.3	1.1	6.6	0.34
221	hypoxia up-regulated protein 1	111.0	7.7	14.9	30.4	1.1	5.9	0.34
222	UDP-glucose:glycoprotein glucosyltransferase 1	185.0	15.6	28.2	47.0	1.1	4.8	0.25
223	very low-density lipoprotein receptor	94.0	4.4	12.5	25.2	1.1	8.6	0.34
224	F-actin-capping protein subunit alpha-2	33.0	3.7	10.1	3.2	1.1	3.5	-0.52
225	coiled-coil domain-containing protein 47 isoform 1	56.0	3.9	8.9	13.4	1.1	5.8	0.20
226	fatty acyl-CoA reductase 1 isoform 1	59.0	1.2	15.6	7.8	1.1	19.3	-0.33
227	NAD(P)H:quinone oxidoreductase type 3, polypeptide A2	13.0	2.4	2.8	2.3	1.1	2.2	-0.09
228	junction plakoglobin isoform 10	82.0	5.4	14.1	17.1	1.1	5.8	0.10
229	transmembrane protein 120A	48.0	2.4	5.8	11.8	1.0	7.4	0.34
230	long-chain-fatty-acid--CoA ligase 3 isoform 2	80.0	2.3	11.0	18.1	1.0	12.8	0.25
231	actin-related protein 2/3 complex subunit 4	20.0	2.6	7.2	0.0	1.0	2.8	-1.00
232	ATPase ASNA1 isoform 1	39.0	1.9	1.7	11.7	1.0	7.0	0.74
233	perilipin-3	48.0	5.6	6.2	10.4	1.0	3.0	0.25
234	ES1 protein homolog, mitochondrial	32.0	4.4	10.7	0.0	0.9	2.4	-1.00
235	uncharacterized glycosyltransferase AGO61 precursor	66.0	2.8	5.7	16.3	0.9	7.9	0.48

236		dystrophin	426.0	31.8	57.3	80.3	0.9	4.3	0.17
237		neutral alpha-glucosidase AB isoform 2	106.0	7.8	10.9	22.2	0.9	4.2	0.34
238		peptidyl-prolyl cis-trans isomerase FKBP8	45.0	3.9	6.2	7.6	0.9	3.6	0.10
239		sec1 family domain-containing protein 1 isoform 2	70.0	4.5	3.6	17.9	0.9	4.7	0.67
240		spectrin beta chain, erythrocyte	247.0	7.5	47.3	28.4	0.9	10.2	-0.25
241		semaphorin-3C	85.0	2.3	20.1	5.9	0.9	11.1	-0.54
242		protein kinase C delta-binding protein	28.0	1.2	4.1	4.4	0.8	7.3	0.04
243		malonyl-CoA decarboxylase, mitochondrial	55.0	1.2	11.7	4.9	0.8	13.7	-0.41
244		serine/threonine-protein phosphatase PP1-beta catalytic subunit	37.0	2.3	1.0	10.1	0.8	4.8	0.81
245		profilin-1-like	18.0	1.3	4.0	1.4	0.8	4.2	-0.48
246		GPI transamidase component PIG-T isoform 2	66.0	1.0	5.7	14.0	0.8	19.5	0.42
247		integrin alpha-7 isoform 2	124.0	6.8	20.0	16.9	0.8	5.4	-0.09
248		peroxiredoxin-6	25.0	3.2	5.9	1.4	0.8	2.3	-0.61
249	rab	ras-related protein Rab-3A	25.0	1.5	5.8	1.4	0.8	4.9	-0.60
250		phosphatidylinositide phosphatase SAC1 isoform 1	67.0	2.9	8.4	11.0	0.8	6.7	0.13
251		talin-2 isoform 1	272.0	0.9	38.4	38.9	0.8	89.1	0.01
252		long-chain fatty acid transport protein 4	72.0	3.0	8.7	11.6	0.8	6.7	0.14
253		amyloid beta A4 protein precursor	87.0	4.5	10.8	13.4	0.8	5.4	0.10
254		chitinase domain-containing protein 1	45.0	1.9	3.5	8.6	0.8	6.3	0.43
255		heme oxygenase 1	33.0	1.2	1.9	7.0	0.7	7.5	0.58
256		ATPase family AAA domain-containing protein 1	41.0	1.2	8.8	2.2	0.7	9.4	-0.60
257		integrin alpha-V isoform 2	109.0	8.6	10.1	18.9	0.7	3.4	0.31
258		endonuclease domain-containing 1 protein	46.0	3.1	4.3	8.0	0.7	4.0	0.30
259		adenylate kinase isoenzyme 1	23.0	2.0	6.0	0.0	0.7	2.9	-1.00
260		voltage-dependent calcium channel subunit alpha-2/delta-1	120.0	10.4	21.9	9.3	0.7	3.0	-0.40
261		endoplasmic reticulum metallopeptidase 1	91.0	4.8	9.0	14.6	0.7	5.0	0.24
262		aldehyde dehydrogenase, mitochondrial isoform 1	51.0	5.3	13.2	0.0	0.7	2.5	-1.00
263		guanine nucleotide-binding protein G(I)/G(S)/G(T) subunit beta-3	37.0	4.7	6.1	3.4	0.7	2.0	-0.28
264		guanine nucleotide-binding protein G(I)/G(S)/G(T) subunit beta-2 isoform 1	37.0	4.7	5.0	4.5	0.7	2.0	-0.05
265		lanosterol synthase isoform 2	83.0	2.3	8.7	12.4	0.7	9.3	0.17
266		atlastin-2-like isoform 1	66.0	1.0	4.4	12.4	0.7	16.6	0.48
267		guanine nucleotide-binding protein G(q) subunit alpha	35.0	3.5	3.5	5.2	0.7	2.5	0.20
268		syntaxin-4 isoform 2	34.0	1.2	5.0	3.4	0.7	7.2	-0.19
269		wolframin	99.0	2.5	10.5	13.1	0.7	9.5	0.11
270		transmembrane anterior posterior transformation protein 1	64.0	1.9	4.7	10.4	0.7	7.8	0.38
271		pre-B-cell leukemia transcription factor-interacting protein 1	78.0	3.3	9.7	8.7	0.7	5.5	-0.05
272		sterol-4-alpha-carboxylate 3-dehydrogenase, decarboxylating isoform 2	42.0	1.2	3.2	6.6	0.7	8.4	0.35
273		protein transport protein Sec61 subunit alpha isoform 2	61.0	1.2	4.3	9.9	0.7	12.2	0.39
274		torsin-1A	38.0	1.2	3.4	5.3	0.6	7.0	0.23
275		CDK5 regulatory subunit-associated protein 3	57.0	1.0	4.6	8.3	0.6	12.8	0.28
276		signal recognition particle receptor subunit alpha-like	70.0	3.0	5.2	10.6	0.6	5.2	0.35
277		enoyl-CoA hydratase domain-containing protein 1 isoform 1	33.0	1.6	7.5	0.0	0.6	4.7	-1.00
278		beta-1-syntrophin isoform 1	57.0	3.9	7.7	5.1	0.6	3.3	-0.21
279		protein ETHE1, mitochondrial	28.0	1.5	6.2	0.0	0.6	4.2	-1.00
280		GDH/6PGL endoplasmic bifunctional protein	89.0	0.8	8.5	11.0	0.6	25.8	0.13
281		dystrobrevin alpha isoform 2	58.0	2.9	5.8	6.7	0.6	4.4	0.07
282		galectin-9	36.0	1.2	5.1	2.3	0.6	6.3	-0.38
283		glucosidase 2 subunit beta	60.0	1.5	3.8	8.4	0.6	8.0	0.38
284		FUN14 domain-containing protein 2	20.0	1.3	2.7	1.4	0.6	3.2	-0.31
285		ERO1-like protein alpha	54.0	1.9	3.3	7.6	0.6	5.6	0.40
286		KDEL motif-containing protein 2	59.0	1.0	4.4	7.5	0.6	12.3	0.26
287		glycerophosphodiester phosphodiesterase 1 isoform 1	38.0	1.2	2.1	5.5	0.6	6.6	0.44
288		extended synaptotagmin-2	96.0	3.3	6.3	12.6	0.6	5.7	0.33
289		tumor protein D54	22.0	1.2	2.8	1.5	0.5	3.6	-0.30
290		3-hydroxyisobutyryl-CoA hydrolase, mitochondrial	40.0	1.2	6.6	1.1	0.5	6.7	-0.71
291		adipocyte plasma membrane-associated protein	46.0	1.1	3.7	5.2	0.5	8.0	0.17
292		acylpyruvase FAHD1, mitochondrial	25.0	1.5	4.8	0.0	0.5	3.3	-1.00
293		clusterin precursor	52.0	2.3	4.3	5.5	0.5	4.2	0.12
294		dolichyl-diphosphooligosaccharide--protein glycosyltransferase subunit ST	94.0	0.8	7.3	10.4	0.5	23.3	0.18
295		beta-2-syntrophin	58.0	2.9	6.2	4.2	0.5	3.6	-0.19

296	FERM domain-containing protein 5	65.0	1.2	6.8	4.6	0.5	9.2	-0.19
297	alpha-sarcoglycan	49.0	2.4	3.5	4.9	0.5	3.5	0.17
298	proactivator polypeptide isoform 6	58.0	1.3	3.4	6.4	0.5	7.6	0.31
299	Golgi pH regulator isoform 2	53.0	1.2	0.7	8.2	0.5	7.1	0.85
300	cGMP-inhibited 3',5'-cyclic phosphodiesterase A-like	137.0	1.7	5.8	16.9	0.5	13.2	0.49
301	desmoplakin	332.0	1.8	14.3	36.4	0.4	28.2	0.44
302	heat shock protein HSP 90-alpha isoform 1	78.0	5.7	10.0	1.7	0.4	2.1	-0.71
303	gelsolin isoform 5	81.0	2.3	6.9	5.0	0.4	5.1	-0.16
304	ankyrin-1	208.0	3.4	29.2	0.0	0.4	8.6	-1.00
305	laminin subunit gamma-1 isoform 1	176.0	7.6	17.9	6.5	0.4	3.2	-0.47
306	lipoprotein lipase isoform 1	53.0	2.1	2.7	4.6	0.4	3.5	0.26
307	epimerase family protein SDR39U1	31.0	1.2	1.9	2.3	0.4	3.5	0.11
308	extended synaptotagmin-1	123.0	5.2	7.0	9.2	0.4	3.2	0.13
309	kinectin	151.0	8.7	4.3	14.5	0.3	2.2	0.54
310	nucleobindin-1 isoform 1	53.0	1.9	1.5	5.1	0.3	3.4	0.55
311	NAD-dependent deacetylase sirtuin-5	34.0	1.2	1.9	2.3	0.3	3.5	0.11
312	golgi-associated plant pathogenesis-related protein 1-like	22.0	1.3	2.7	0.0	0.3	2.1	-1.00
313	long-chain-fatty-acid--CoA ligase 6 isoform 3	78.0	3.8	2.2	7.1	0.3	2.5	0.53
314	armadillo repeat-containing X-linked protein 3 isoform 1	42.0	1.2	3.9	1.1	0.3	4.3	-0.56
315	sideroflexin-3	36.0	1.2	3.1	1.1	0.3	3.7	-0.47
316	transducin (beta)-like 2 isoform 6	50.0	1.1	1.5	4.3	0.3	5.2	0.49
317	Golgi apparatus protein 1 isoform 1	135.0	3.4	7.2	8.2	0.3	4.6	0.07
318	mannosyl-oligosaccharide glucosidase	92.0	0.8	6.2	4.2	0.3	12.6	-0.19
319	ATP-binding cassette sub-family D member 2 isoform 1	84.0	3.5	4.5	4.8	0.3	2.6	0.03
320	alpha-actinin-4 isoform 1	107.0	5.8	11.8	0.0	0.3	2.0	-1.00
321	isobutyryl-CoA dehydrogenase, mitochondrial	50.0	2.3	5.3	0.0	0.3	2.3	-1.00
322	spectrin alpha chain, erythrocyte	273.0	1.7	27.1	1.5	0.3	16.9	-0.90
323	ryanodine receptor 3	555.0	25.1	41.7	15.7	0.3	2.3	-0.45
324	talin-1-like	270.0	5.1	19.7	7.5	0.3	5.4	-0.45
325	filamin-A isoform 2	281.0	6.7	14.8	13.5	0.3	4.2	-0.04
326	laminin subunit alpha-2	342.0	6.5	20.5	13.5	0.3	5.2	-0.21
327	procollagen-lysine,2-oxoglutarate 5-dioxygenase 1 isoform 1	84.0	1.5	3.9	4.2	0.3	5.3	0.05
328	E3 UFM1-protein ligase 1	89.0	1.5	3.3	5.1	0.3	5.6	0.21
329	cadherin-2 isoform 2	100.0	3.6	5.5	2.6	0.2	2.3	-0.36
330	complement C3	181.0	1.8	7.5	6.0	0.2	7.7	-0.11
331	melanoma inhibitory activity protein 3-like	206.0	1.1	4.2	11.2	0.2	14.1	0.46
332	protein phosphatase 1 regulatory subunit 3A	126.0	0.9	3.0	6.4	0.2	10.8	0.37
333	integrin beta-6 isoform 3	88.0	0.8	1.7	4.4	0.2	7.3	0.45
334	procollagen-lysine,2-oxoglutarate 5-dioxygenase 3 isoform 1	85.0	0.8	1.5	4.2	0.2	7.6	0.46
335	BAG family molecular chaperone regulator 3	62.0	1.5	0.7	3.3	0.2	2.6	0.63
336	monocarboxylate transporter 1 isoform 1	54.0	1.0	0.9	2.6	0.2	3.6	0.50
337	fibrillin-1 isoform 3	312.0	1.1	9.5	9.9	0.2	17.8	0.02
338	titin	3725.0	39.6	72.0	153.5	0.2	5.7	0.36
339	nebulette isoform 3	116.0	0.9	2.6	4.3	0.2	8.1	0.24
340	laminin subunit beta-2	196.0	0.9	9.3	2.3	0.2	13.4	-0.60
341	actin-related protein 2 isoform 1	45.0	1.2	2.6	0.0	0.2	2.2	-1.00
342	protein sel-1 homolog 1	89.0	0.8	1.6	3.4	0.2	6.0	0.36
343	mitofusin-1	84.0	1.5	3.9	0.8	0.2	3.1	-0.64
344	IQ motif containing GTPase activating protein 1	189.0	1.7	9.9	0.0	0.1	5.7	-1.00
345	heat shock 70 kDa protein 12B	75.0	1.5	3.7	0.0	0.1	2.4	-1.00
346	laminin subunit beta-1 isoform 1	198.0	0.9	5.8	3.1	0.1	10.4	-0.30
347	selenium-binding protein 1	67.0	1.2	2.6	0.0	0.1	2.1	-1.00
348	basement membrane-specific heparan sulfate proteoglycan core protein	471.0	5.4	12.4	4.9	0.1	3.2	-0.44
349	sortilin	77.0	0.8	0.8	1.8	0.1	3.2	0.36
350	RAD50-interacting protein 1	130.0	0.8	1.5	2.5	0.1	5.4	0.24
351	von Willebrand factor precursor	310.0	0.8	9.7	0.0	0.1	11.7	-1.00
352	protocadherin-7	137.0	0.9	0.8	2.4	0.1	3.7	0.51
353	coagulation factor XIII A chain isoform 1	83.0	0.8	1.5	0.0	0.1	2.0	-1.00
354	cytoplasmic FMR1-interacting protein 1	146.0	0.9	1.6	0.8	0.0	2.8	-0.30

496 proteins not meeting enrichment criterion for SR enriched (i.e., ESR values ≥2.0). Proteins listed in order of decreasing ASR

cardiac actin, alpha-1	37.0	162.7	153.0	159.0	23.5	1.9	0.02
malate dehydrogenase, mitochondrial	35.0	149.2	194.4	47.4	19.2	1.6	-0.61
voltage-dependent anion-selective channel protein-1	31.0	141.2	133.6	75.4	18.8	1.5	-0.28
ATP synthase beta-1 subunit	56.0	645.0	251.5	111.4	18.0	0.6	-0.39
keratin, type I cytoskeletal-10	58.0	259.4	150.3	224.0	18.0	1.4	0.20
ADP/ATP translocase 1	33.0	410.6	146.4	51.1	16.7	0.5	-0.48
voltage-dependent anion-selective channel protein 2	32.0	129.6	106.5	70.4	15.4	1.4	-0.20
ATP synthase, alpha-2 subunit	60.0	735.0	269.3	59.5	15.3	0.4	-0.64
keratin, type II cytoskeletal 1	64.0	212.5	134.2	195.0	14.3	1.5	0.18
serum albumin	69.0	240.8	164.5	183.3	14.0	1.4	0.05
keratin, type II cytoskeletal 78	56.0	168.2	109.4	132.6	12.0	1.4	0.10
Phosphate carrier protein-5	40.0	325.4	123.3	41.8	11.5	0.5	-0.49
NADH dehydrogenase beta subcomplex-10	21.0	110.1	69.2	12.3	10.8	0.7	-0.70
keratin, type II cytoskeletal 5	63.0	131.6	92.7	146.0	10.6	1.8	0.22
actin, alpha skeletal muscle isoform 2	42.0	93.9	72.2	77.1	9.9	1.6	0.03
ATP synthase subunit O, mitochondrial isoform 1	23.0	76.0	52.2	27.9	9.7	1.1	-0.30
actin, cytoplasmic 1	42.0	80.8	68.9	77.3	9.7	1.8	0.06
keratin, type II cytoskeletal 73	65.0	135.4	90.3	126.3	9.3	1.6	0.17
keratin 14	52.0	81.0	54.6	98.7	8.2	1.9	0.29
keratin, type I cytoskeletal 42	51.0	86.4	57.4	83.7	7.7	1.6	0.19
long-chain-fatty-acid--CoA ligase 1 isoform 5	78.0	127.3	90.0	117.6	7.4	1.6	0.13
cytochrome c oxidase subunit 5A	17.0	43.4	32.2	12.6	7.3	1.0	-0.44
hemoglobin subunit beta-like isoform 2	18.0	25.8	28.5	18.7	7.3	1.8	-0.21
aspartate aminotransferase	47.0	106.1	77.7	45.3	7.3	1.2	-0.26
annexin A2	39.0	78.9	67.5	33.0	7.2	1.3	-0.34
cytochrome c oxidase subunit 4 isoform 1	20.0	51.8	33.8	15.4	6.9	0.9	-0.37
ATP synthase subunit d, mitochondrial-like	19.0	33.2	33.8	12.6	6.8	1.4	-0.46
keratin, type II cytoskeletal 75	58.0	88.9	51.2	86.3	6.6	1.5	0.26
ATP synthase subunit b	29.0	111.6	50.9	17.7	6.6	0.6	-0.48
voltage-dependent anion-selective channel protein 3 isoform 1	31.0	55.3	43.2	30.0	6.6	1.3	-0.18
NADH dehydrogenase [ubiquinone] flavoprotein 2	27.0	68.7	44.2	19.0	6.5	0.9	-0.40
caveolin-3	17.0	20.0	18.6	19.1	6.2	1.9	0.01
myoglobin	17.0	19.3	22.6	14.0	6.0	1.9	-0.23
cytochrome c1, heme protein, mitochondrial isoform 1	35.0	130.6	64.2	10.1	5.9	0.6	-0.73
keratin, type II cytoskeletal 2 epidermal	65.0	107.8	52.3	85.1	5.9	1.3	0.24
creatine kinase M-type	43.0	71.0	56.6	32.8	5.8	1.3	-0.27
cytochrome b-c1 complex subunit Rieske	29.0	64.8	45.1	14.7	5.7	0.9	-0.51
pyruvate dehydrogenase E1 component subunit beta, mitochondrial isofor	39.0	139.1	58.5	16.2	5.3	0.5	-0.57
NADH dehydrogenase [ubiquinone] 1 beta subcomplex subunit 7	16.0	30.4	22.6	7.0	5.1	1.0	-0.53
NADH dehydrogenase [ubiquinone] 1 alpha subcomplex subunit 4-like	9.0	18.3	13.3	2.8	5.0	0.9	-0.65
myosin light chain 3 isoform 1	25.0	79.1	24.3	20.4	5.0	0.6	-0.09
ATP synthase subunit gamma, mitochondrial isoform 3	33.0	75.3	44.6	13.5	4.9	0.8	-0.53
sodium/potassium-transporting ATPase subunit alpha-3	129.0	252.4	117.6	107.0	4.9	0.9	-0.05
cytochrome c	12.0	25.9	10.6	9.8	4.7	0.8	-0.04
cytochrome c oxidase subunit II	26.0	76.5	32.0	11.6	4.7	0.6	-0.47
NADH dehydrogenase [ubiquinone] iron-sulfur protein 3, mitochondrial	30.0	55.5	34.4	14.4	4.5	0.9	-0.41
ecto-ADP-ribosyltransferase 3 isoform 2	43.0	39.8	41.0	28.6	4.5	1.7	-0.18
CD81 protein	27.0	21.7	27.2	12.9	4.1	1.8	-0.36
keratin, type I cytoskeletal 16	52.0	43.1	31.7	45.1	4.1	1.8	0.17
very long-chain specific acyl-CoA dehydrogenase, mitochondrial isoform 1	70.0	161.7	79.3	22.3	4.0	0.6	-0.56
hemoglobin subunit alpha-like	15.0	12.9	10.6	9.8	3.8	1.6	-0.04
microsomal glutathione S-transferase 3	17.0	25.5	18.7	4.3	3.8	0.9	-0.63
superoxide dismutase [Mn], mitochondrial isoform 1	25.0	25.0	30.1	3.6	3.8	1.3	-0.79
electron transfer flavoprotein subunit beta isoform 1	28.0	22.2	33.4	4.3	3.8	1.7	-0.77
mitochondrial 2-oxoglutarate/malate carrier protein	33.0	87.0	24.7	17.3	3.5	0.5	-0.18
succinate dehydrogenase [ubiquinone] iron-sulfur subunit	32.0	62.6	30.1	10.1	3.5	0.6	-0.50
NADH dehydrogenase [ubiquinone] 1 alpha subcomplex subunit 13 isoform	19.0	25.1	17.8	5.6	3.4	0.9	-0.52
cytochrome b-c1 complex subunit 2, mitochondrial isoform 1	48.0	121.6	42.1	15.6	3.3	0.5	-0.46
cytochrome c oxidase subunit 6B1-like isoform 1	10.0	12.9	11.9	0.0	3.3	0.9	-1.00
calmodulin isoform 2	17.0	12.3	10.4	9.8	3.3	1.6	-0.03

ATP synthase subunit g, mitochondrial-like isoform 1	11.0	13.0	10.1	2.8	3.3	1.0	-0.56
medium-chain specific acyl-CoA dehydrogenase, mitochondrial	49.0	48.9	46.8	10.4	3.2	1.2	-0.64
cytochrome c oxidase polypeptide Vb	14.0	18.7	10.6	5.6	3.2	0.9	-0.31
cytochrome b-c1 complex subunit 7-like isoform 2	14.0	24.5	13.3	2.8	3.2	0.7	-0.65
60S ribosomal protein L24 isoform 2	18.0	12.5	12.6	7.8	3.2	1.6	-0.24
NADH dehydrogenase [ubiquinone] 1 beta subcomplex subunit 9	22.0	22.7	24.6	0.0	3.1	1.1	-1.00
NADH dehydrogenase [ubiquinone] iron-sulfur protein 7, mitochondrial	24.0	44.5	18.8	7.4	3.0	0.6	-0.44
sodium/potassium-transporting ATPase subunit beta-1	35.0	28.3	15.2	22.3	3.0	1.3	0.19
enoyl-CoA hydratase, mitochondrial	31.0	28.9	23.2	10.1	3.0	1.2	-0.39
annexin A6 isoform 2	76.0	89.4	47.3	31.2	2.9	0.9	-0.20
NADH dehydrogenase [ubiquinone] 1 alpha subcomplex subunit 2 isoform	11.0	12.9	8.5	2.8	2.9	0.9	-0.50
fructose-bisphosphate aldolase A isoform 2	40.0	23.8	25.6	13.8	2.7	1.7	-0.30
triosephosphate isomerase	27.0	17.8	14.6	11.5	2.7	1.5	-0.12
keratin, type I cytoskeletal 24	56.0	32.7	19.8	34.3	2.7	1.7	0.27
sodium/potassium-transporting ATPase subunit alpha-1 precursor	113.0	126.1	59.5	49.0	2.7	0.9	-0.10
prohibitin	30.0	42.7	19.9	8.6	2.6	0.7	-0.39
trifunctional enzyme subunit alpha, mitochondrial-like	83.0	156.6	52.8	24.9	2.6	0.5	-0.36
ras-related protein Rab-5A	24.0	11.6	12.0	10.2	2.6	1.9	-0.08
cardiac myosin regulatory light chain 2	19.0	64.7	11.3	6.0	2.5	0.3	-0.31
NADH dehydrogenase [ubiquinone] 1 subunit C2 isoform 7	14.0	11.7	9.8	2.8	2.5	1.1	-0.56
glyceraldehyde-3-phosphate dehydrogenase	36.0	30.2	21.0	11.4	2.5	1.1	-0.29
creatine kinase S-type, mitochondrial precursor	47.0	88.5	26.6	15.6	2.5	0.5	-0.26
NADH-ubiquinone oxidoreductase 75 kDa subunit	74.0	208.5	51.8	14.5	2.5	0.3	-0.56
cytochrome b-c1 complex subunit 1, mitochondrial isoform 2	53.0	92.0	34.9	12.5	2.5	0.5	-0.47
pyruvate dehydrogenase E1 component subunit alpha, somatic form	44.0	145.0	34.0	5.2	2.5	0.3	-0.74
3-ketoacyl-CoA thiolase, mitochondrial	36.0	27.3	28.3	3.5	2.5	1.2	-0.78
guanine nucleotide-binding protein G(o) subunit alpha isoform 1	40.0	24.6	19.2	15.9	2.4	1.4	-0.09
ras-related C3 botulinum toxin substrate 1 precursor	21.0	14.4	18.1	0.0	2.4	1.3	-1.00
60 kDa heat shock protein, mitochondrial isoform 1	60.0	30.0	42.6	9.1	2.4	1.7	-0.65
ADP-ribosylation factor-like 8B	22.0	12.6	11.7	6.9	2.4	1.5	-0.26
NADH dehydrogenase [ubiquinone] iron-sulfur protein 8, mitochondrial	24.0	26.3	15.3	4.9	2.3	0.8	-0.51
heat shock cognate 71 kDa protein isoform 1	71.0	43.8	24.1	34.8	2.3	1.3	0.18
ras-related protein Rap-1A isoform 2	21.0	11.5	15.4	1.8	2.3	1.5	-0.79
coiled-coil-helix-coiled-coil-helix domain-containing protein 3, mito form 2	26.0	18.0	13.5	7.2	2.2	1.1	-0.30
guanine nucleotide-binding protein G(i) subunit alpha-2	41.0	19.5	19.8	12.3	2.2	1.6	-0.23
guanine nucleotide-binding protein G(s) subunit alpha	46.0	25.2	19.0	16.9	2.2	1.4	-0.06
mitochondrial carrier homolog 2 isoform 6	33.0	17.7	18.7	7.0	2.2	1.5	-0.46
cytochrome b-c1 complex subunit 9 isoform 2	7.0	5.1	4.0	1.4	2.1	1.0	-0.48
ADP-ribosylation factor 1	21.0	11.0	10.4	5.6	2.1	1.5	-0.30
cytochrome c oxidase subunit 7A1	9.0	13.0	5.3	1.4	2.1	0.5	-0.58
14-3-3 protein epsilon isoform 2	29.0	15.1	13.5	7.8	2.0	1.4	-0.27
fatty acid-binding protein, heart isoform 1	15.0	7.7	4.0	7.0	2.0	1.4	0.28
NAD(P) transhydrogenase, mitochondrial isoform 1	114.0	390.9	68.5	14.9	2.0	0.2	-0.64
NADH dehydrogenase [ubiquinone] 1 beta subcomplex subunit 4	15.0	9.0	9.5	1.4	2.0	1.2	-0.74
thioredoxin-dependent peroxide reductase, mitochondrial	28.0	10.9	18.7	1.5	2.0	1.9	-0.85
60S ribosomal protein L18	22.0	30.0	15.5	0.0	2.0	0.5	-1.00
aconitate hydratase, mitochondrial isoform 3	86.0	51.0	53.5	6.8	2.0	1.2	-0.78
annexin A11	54.0	35.9	21.8	15.1	1.9	1.0	-0.18
glyceraldehyde-3-phosphate dehydrogenase, testis-specific	44.0	22.7	20.2	9.7	1.9	1.3	-0.35
synaptojanin-2-binding protein	16.0	6.4	6.6	4.2	1.9	1.7	-0.22
adiponectin precursor	26.0	13.1	8.1	9.5	1.9	1.3	0.08
trifunctional enzyme subunit beta, mitochondrial isoform 1	51.0	45.2	22.8	11.6	1.9	0.8	-0.33
prohibitin-2	33.0	24.5	15.0	6.8	1.8	0.9	-0.38
40S ribosomal protein S9	23.0	20.1	14.4	0.0	1.7	0.7	-1.00
calcium-binding mitochondrial carrier protein Aralar1 isoform 1	75.0	105.6	32.5	13.9	1.7	0.4	-0.40
methylglutaconyl-CoA hydratase, mitochondrial	36.0	32.8	15.9	5.8	1.7	0.7	-0.47
myosin-7	223.0	655.7	74.1	58.5	1.7	0.2	-0.12
60S ribosomal protein L10-like	17.0	10.8	8.2	1.8	1.6	0.9	-0.64
electron transfer flavoprotein subunit alpha, mitochondrial isoform 1	35.0	13.0	17.0	3.5	1.6	1.6	-0.66
succinyl-CoA ligase [ADP-forming] subunit beta, mitochondrial	44.0	26.9	18.6	6.9	1.6	0.9	-0.46

acetyl-CoA acetyltransferase, mitochondrial	46.0	30.3	17.6	8.6	1.6	0.9	-0.34
carnitine O-palmitoyltransferase 1, muscle isoform isoform 1	88.0	65.6	34.7	15.3	1.6	0.8	-0.39
ADP/ATP translocase 2 isoform 2	33.0	50.1	13.9	4.3	1.5	0.4	-0.53
60S ribosomal protein L13	24.0	8.9	8.8	4.3	1.5	1.5	-0.34
NADH dehydrogenase [ubiquinone] 1 beta subcomplex subunit 5, mito forn	22.0	14.8	10.6	1.4	1.5	0.8	-0.77
cytochrome b-c1 complex subunit 8	10.0	3.9	4.0	1.4	1.5	1.4	-0.48
annexin A5	36.0	22.3	13.3	5.7	1.5	0.9	-0.40
ras-related protein Rab-31	22.0	13.3	9.8	1.8	1.5	0.9	-0.69
NADH dehydrogenase [ubiquinone] 1 beta subcomplex subunit 8, mito forn	22.0	13.0	11.3	0.0	1.4	0.9	-1.00
ferritin light chain	20.0	5.9	10.2	0.0	1.4	1.7	-1.00
cathepsin D precursor	44.0	15.9	11.6	10.7	1.4	1.4	-0.04
NADH dehydrogenase [ubiquinone] iron-sulfur protein 2, mitochondrial isof	52.0	97.3	20.9	5.2	1.4	0.3	-0.60
keratin, type II cytoskeletal 8	55.0	15.3	0.0	27.3	1.4	1.8	1.00
keratin, type II cytoskeletal 4	64.0	30.3	8.1	23.5	1.4	1.0	0.49
cadherin-13 isoform 2	78.0	67.7	19.3	18.7	1.4	0.6	-0.02
cysteine and glycine-rich protein 3 isoform 5	20.0	13.9	7.2	2.3	1.3	0.7	-0.52
pyruvate kinase isozymes M1/M2 isoform 1	58.0	24.1	20.0	7.6	1.3	1.1	-0.45
isocitrate dehydrogenase [NAD] subunit gamma, mitochondrial isoform 1	43.0	13.1	12.9	7.4	1.3	1.5	-0.27
cardiac troponin I	24.0	37.2	6.9	4.3	1.3	0.3	-0.23
syntaxin-7 isoform 5	30.0	13.1	8.6	5.4	1.3	1.1	-0.23
OCIA domain-containing protein 1 isoform 2	28.0	6.5	8.0	5.0	1.3	2.0	-0.23
carnitine O-acetyltransferase isoform 1	71.0	36.6	24.0	8.3	1.3	0.9	-0.49
mitochondrial inner membrane protein isoform 7	83.0	43.2	31.2	6.4	1.3	0.9	-0.66
39S ribosomal protein L12, mitochondrial	21.0	18.2	9.4	0.0	1.2	0.5	-1.00
cAMP-dependent protein kinase type II-alpha regulatory subunit isoform 1	45.0	17.4	12.7	7.4	1.2	1.2	-0.26
synaptosomal-associated protein 23 isoform 2	23.0	5.9	7.2	2.9	1.2	1.7	-0.43
electron transfer flavoprotein-ubiquinone oxidoreductase, mitochondrial isc	69.0	53.2	24.2	5.8	1.2	0.6	-0.61
NADH dehydrogenase [ubiquinone] 1 alpha subcomplex subunit 8	20.0	9.7	5.9	2.8	1.2	0.9	-0.35
glycogen phosphorylase, muscle form isoform 1	97.0	184.7	35.9	6.2	1.2	0.2	-0.71
mitochondrial carrier homolog 1 isoform 2	31.0	16.9	8.6	4.8	1.2	0.8	-0.28
synaptogyrin 2	25.0	7.1	4.8	5.9	1.2	1.5	0.10
60S ribosomal protein L27a-like	17.0	6.6	5.9	1.4	1.2	1.1	-0.61
ubiquinone biosynthesis protein COQ9, mitochondrial isoform 1	36.0	9.7	5.3	10.0	1.2	1.6	0.31
isocitrate dehydrogenase [NADP], mitochondrial	77.0	16.6	26.4	6.0	1.2	2.0	-0.63
NADH dehydrogenase [ubiquinone] 1 alpha subcomplex subunit 5	13.0	9.1	4.0	1.4	1.2	0.6	-0.48
protein NipSnap homolog 2 isoform 1	34.0	34.2	9.8	4.3	1.2	0.4	-0.39
succinate dehydrogenase [ubiquinone] flavoprotein subunit, mito form 1	73.0	95.9	19.4	10.7	1.1	0.3	-0.29
NADH dehydrogenase [ubiquinone] 1 alpha subcomplex subunit 9, mito for	43.0	24.7	13.2	4.5	1.1	0.7	-0.49
elongation factor Tu, mitochondrial	49.0	11.7	13.9	6.0	1.1	1.7	-0.40
transmembrane protein 65	26.0	26.5	10.4	0.0	1.1	0.4	-1.00
stress-70 protein, mitochondrial isoform 6	74.0	35.6	24.2	4.8	1.1	0.8	-0.67
40S ribosomal protein S3 isoform 1	27.0	35.2	8.3	2.3	1.1	0.3	-0.56
fructose-bisphosphate aldolase C isoform 1	39.0	15.9	13.0	2.2	1.1	1.0	-0.71
mitochondrial fission process protein 1	18.0	11.7	7.0	0.0	1.1	0.6	-1.00
apoptosis-inducing factor 1, mitochondrial isoform 6	66.0	69.2	19.4	6.2	1.1	0.4	-0.52
calcium-binding mitochondrial carrier protein Aralar2	74.0	56.0	22.4	6.0	1.1	0.5	-0.57
coenzyme Q-binding protein COQ10 homolog A, mitochondrial	27.0	8.5	10.2	0.0	1.1	1.2	-1.00
NADH dehydrogenase subunit 1	36.0	46.3	12.1	1.4	1.0	0.3	-0.79
citrate synthase, mitochondrial isoform 1	51.0	28.8	12.8	5.4	1.0	0.6	-0.41
NADH dehydrogenase [ubiquinone] 1 alpha subcomplex subunit 6	23.0	10.3	6.6	1.4	1.0	0.8	-0.65
muscle-related coiled-coil protein	42.0	19.9	10.0	4.6	1.0	0.7	-0.37
guanine nucleotide-binding protein G(I)/G(S)/G(T) subunit beta-1	37.0	7.1	7.3	5.4	1.0	1.8	-0.15
dihydrolipoyllysine-residue acetyltransferase component of PDH complex, r	69.0	67.7	18.5	4.8	0.9	0.3	-0.59
poly(rC)-binding protein 1 isoform 1	37.0	7.8	6.0	6.3	0.9	1.6	0.02
dihydrolipoyllysine-residue succinyltransferase comp of 2-oxoglutarate DH	49.0	51.3	13.2	2.9	0.9	0.3	-0.64
60S ribosomal protein L12	18.0	17.6	5.7	0.0	0.9	0.3	-1.00
NADH dehydrogenase [ubiquinone] 1 alpha subcomplex subunit 10, mitoch	40.0	23.0	10.7	1.7	0.9	0.5	-0.72
collagen alpha-1(VI) chain	109.0	36.9	20.7	12.7	0.9	0.9	-0.24
glutathione S-transferase pi 1	24.0	4.2	5.8	1.5	0.8	1.7	-0.59
D-beta-hydroxybutyrate dehydrogenase, mitochondrial	39.0	26.2	10.6	1.2	0.8	0.4	-0.80

apolipoprotein O-like	29.0	5.6	8.7	0.0	0.8	1.6	-1.00
guanine nucleotide-binding protein G(k) subunit alpha	41.0	10.5	12.2	0.0	0.8	1.2	-1.00
6-phosphofructokinase, muscle type	86.0	19.7	23.1	2.5	0.8	1.3	-0.80
isovaleryl-CoA dehydrogenase, mitochondrial-like isoform 1	46.0	9.1	12.3	1.1	0.8	1.5	-0.83
succinyl-CoA:3-ketoacid-coenzyme A transferase 1, mitochondrial	65.0	25.5	17.2	1.7	0.8	0.7	-0.82
beta-enolase isoform 1	47.0	13.3	8.9	4.7	0.8	1.0	-0.31
sulfide:quinone oxidoreductase, mitochondrial isoform 2	50.0	20.0	9.1	5.3	0.8	0.7	-0.26
NADH dehydrogenase [ubiquinone] flavoprotein 1, mitochondrial isoform 2	51.0	45.0	12.3	2.0	0.8	0.3	-0.73
phosphoglycerate mutase 2 isoform 1	29.0	6.3	3.7	4.3	0.8	1.3	0.07
sorting and assembly machinery component 50 homolog	52.0	16.7	11.5	2.8	0.8	0.9	-0.61
ubiquinone biosynthesis protein COQ7 homolog	24.0	10.0	6.6	0.0	0.8	0.7	-1.00
lectin, galactoside-binding, soluble, 1	15.0	3.9	2.7	1.4	0.8	1.1	-0.31
60S ribosomal protein L18a isoform 1	21.0	6.0	5.7	0.0	0.8	1.0	-1.00
guanine nucleotide-binding protein subunit alpha-11	42.0	8.5	5.2	6.1	0.7	1.3	0.07
branched-chain-amino-acid aminotransferase, mitochondrial isoform 1	34.0	7.0	9.0	0.0	0.7	1.3	-1.00
fumarate hydratase, mitochondrial isoform 1	54.0	14.4	12.5	1.7	0.7	1.0	-0.76
aspartate aminotransferase, cytoplasmic isoform 1	46.0	15.6	7.0	5.2	0.7	0.8	-0.15
annexin A4	36.0	7.1	8.3	1.2	0.7	1.3	-0.75
basal cell adhesion molecule	68.0	11.8	10.6	6.4	0.7	1.4	-0.24
glycogen phosphorylase, brain form	97.0	88.1	20.2	3.5	0.7	0.3	-0.70
short-chain specific acyl-CoA dehydrogenase, mitochondrial	46.0	9.9	9.3	2.0	0.7	1.1	-0.65
40S ribosomal protein S13	17.0	5.1	1.3	2.8	0.7	0.8	0.36
growth hormone-inducible transmembrane protein isoform 3	37.0	33.1	9.0	0.0	0.7	0.3	-1.00
NADH dehydrogenase [ubiquinone] 1 alpha subcomplex subunit 12	17.0	9.0	4.0	0.0	0.7	0.4	-1.00
14-3-3 protein zeta/delta isoform 2	28.0	4.8	4.9	1.4	0.6	1.3	-0.55
phosphatidylethanolamine-binding protein 1	21.0	6.0	4.8	0.0	0.6	0.8	-1.00
ribosomal protein, large, P0	34.0	10.0	4.6	3.2	0.6	0.8	-0.18
dysferlin isoform 9	241.0	37.5	31.3	23.3	0.6	1.5	-0.15
coiled-coil domain-containing protein 56	12.0	3.9	2.7	0.0	0.6	0.7	-1.00
60S acidic ribosomal protein P2	12.0	3.9	2.7	0.0	0.6	0.7	-1.00
glycogen debranching enzyme	175.0	67.1	14.6	23.3	0.6	0.6	0.23
V-type proton ATPase subunit d 1 isoform 1	40.0	5.9	4.3	4.4	0.6	1.5	0.02
cardiac troponin C	18.0	49.6	3.8	0.0	0.6	0.1	-1.00
cofilin-2	19.0	2.6	4.0	0.0	0.6	1.5	-1.00
methylmalonate-semialdehyde dehydrogenase [acylating], mitochondrial	59.0	19.3	10.5	1.8	0.6	0.6	-0.71
armadillo repeat-containing protein 10	27.0	6.0	5.6	0.0	0.6	0.9	-1.00
tropomyosin alpha-4 chain isoform 6	33.0	75.4	5.6	1.1	0.6	0.1	-0.68
heat shock 70 kDa protein 1	70.0	15.4	7.8	6.0	0.6	0.9	-0.13
60S ribosomal protein L7a	30.0	15.9	5.9	0.0	0.5	0.4	-1.00
dihydrolipoyl dehydrogenase, mitochondrial precursor	54.0	38.0	9.8	0.8	0.5	0.3	-0.84
aarF domain containing kinase 3	64.0	9.9	11.7	0.8	0.5	1.3	-0.87
EH domain-containing protein 2	61.0	21.5	7.2	4.8	0.5	0.6	-0.20
brain protein 44	14.0	7.7	2.7	0.0	0.5	0.3	-1.00
NADH dehydrogenase [ubiquinone] 1 alpha subcomplex assembly factor 4	20.0	2.0	3.8	0.0	0.5	1.9	-1.00
EH domain-containing protein 4	61.0	15.1	6.4	4.9	0.5	0.7	-0.14
acyl-CoA dehydrogenase family member 9, mitochondrial isoform 1	69.0	30.2	11.8	0.8	0.5	0.4	-0.87
collagen alpha-2(VI) chain-like	87.0	13.8	11.8	4.2	0.5	1.2	-0.47
uncharacterized protein C18orf19 homolog	31.0	8.5	5.7	0.0	0.5	0.7	-1.00
NADH dehydrogenase subunit 5	68.0	53.0	12.4	0.0	0.5	0.2	-1.00
cytosolic 5'-nucleotidase 3 isoform 2	37.0	3.7	4.6	2.1	0.5	1.8	-0.37
ezrin isoform 1	69.0	15.2	11.3	1.1	0.5	0.8	-0.83
sodium/calcium exchanger 1	104.0	17.9	9.1	9.0	0.5	1.0	0.00
solute carrier family 2, facilitated glucose transporter member 4	55.0	7.5	6.8	2.5	0.5	1.2	-0.46
protein kinase C and casein kinase substrate in neurons protein 3 isoform 1	49.0	7.2	4.3	3.9	0.5	1.1	-0.05
60S ribosomal protein L13a	24.0	5.4	4.0	0.0	0.5	0.7	-1.00
NADH dehydrogenase subunit 2	39.0	39.6	6.4	0.0	0.5	0.2	-1.00
heat shock protein HSP 90-beta isoform 1	83.0	10.4	8.5	5.1	0.5	1.3	-0.25
mitochondrial carnitine/acylcarnitine carrier protein isoform 4	33.0	16.1	5.4	0.0	0.5	0.3	-1.00
protein ERGIC-53	58.0	4.9	1.8	7.6	0.5	1.9	0.62
transitional endoplasmic reticulum ATPase isoform 3	89.0	19.4	6.6	7.6	0.4	0.7	0.07

collagen alpha-3(VI) chain precursor	343.0	65.2	35.6	18.8	0.4	0.8	-0.31
NADH dehydrogenase [ubiquinone] 1 beta subcomplex subunit 11, mitocho	17.0	2.6	2.7	0.0	0.4	1.0	-1.00
uncharacterized protein LOC479805	17.0	5.1	2.7	0.0	0.4	0.5	-1.00
hydroxysteroid (17-beta) dehydrogenase 8 isoform 2	27.0	2.4	3.0	1.2	0.4	1.8	-0.44
MHC class I DLA-12 precursor	40.0	5.0	4.3	1.7	0.4	1.2	-0.43
ragulator complex protein LAMTOR1 isoform 1	18.0	2.6	1.3	1.4	0.4	1.1	0.03
60S ribosomal protein L17-like	21.0	4.0	3.1	0.0	0.4	0.8	-1.00
phosphoglycerate kinase 1 isoform 2	44.0	3.8	4.8	1.7	0.4	1.7	-0.47
protein FAM162A	18.0	2.6	2.7	0.0	0.4	1.0	-1.00
adenylate kinase 2, mitochondrial isoform 2	26.0	13.4	3.7	0.0	0.4	0.3	-1.00
2,4-dienoyl-CoA reductase, mitochondrial	35.0	12.1	5.0	0.0	0.4	0.4	-1.00
cytochrome c oxidase subunit I	57.0	30.4	4.8	3.2	0.4	0.3	-0.19
syntaxin-12	31.0	6.1	3.3	1.1	0.4	0.7	-0.51
14-3-3 protein eta	28.0	4.8	2.5	1.4	0.4	0.8	-0.27
uncharacterized protein LOC477037	10.0	2.6	1.3	0.0	0.4	0.5	-1.00
serotransferrin isoform 1	78.0	20.0	5.9	4.0	0.4	0.5	-0.19
2-oxoglutarate dehydrogenase, mitochondrial	105.0	63.5	13.3	0.0	0.4	0.2	-1.00
28S ribosomal protein S36, mitochondrial	11.0	3.9	1.3	0.0	0.3	0.3	-1.00
elongation factor 1-alpha 1	50.0	29.9	4.1	2.0	0.3	0.2	-0.35
moesin isoform 2	68.0	17.4	7.4	0.8	0.3	0.5	-0.80
translationally-controlled tumor protein isoform 1	20.0	1.4	2.3	0.0	0.3	1.7	-1.00
secretory carrier-associated membrane protein 3 isoform 1	38.0	4.9	2.3	2.1	0.3	0.9	-0.04
ubiquinol-cytochrome c reductase complex chaperone CBP3 homolog isofor	35.0	20.3	4.0	0.0	0.3	0.2	-1.00
mitochondrial fission factor isoform 3	33.0	2.3	2.9	0.9	0.3	1.6	-0.54
annexin A1	39.0	5.0	2.3	2.1	0.3	0.9	-0.04
pantetheinase-like	58.0	9.8	5.0	1.5	0.3	0.7	-0.54
protein lin-7 homolog C	22.0	1.4	2.3	0.0	0.3	1.7	-1.00
alpha-enolase isoform 1	49.0	3.6	5.2	0.0	0.3	1.4	-1.00
60S ribosomal protein L23a-like isoform 1	18.0	9.1	1.9	0.0	0.3	0.2	-1.00
glutamate dehydrogenase 1, mitochondrial	65.0	21.4	6.8	0.0	0.3	0.3	-1.00
solute carrier family 12 member 7	134.0	15.5	10.1	3.8	0.3	0.9	-0.45
40S ribosomal protein S8 isoform 6	24.0	18.3	2.5	0.0	0.3	0.1	-1.00
B-cell receptor-associated protein 31	28.0	1.6	0.0	2.9	0.3	1.8	1.00
uncharacterized protein LOC475739	13.0	3.9	1.3	0.0	0.3	0.3	-1.00
60S ribosomal protein L10a	25.0	6.0	2.5	0.0	0.3	0.4	-1.00
prelamin-A/C isoform 4	74.0	4.5	0.7	6.5	0.3	1.6	0.80
myosin-Ic-like isoform 1	122.0	10.6	10.2	1.7	0.3	1.1	-0.72
carnitine O-palmitoyltransferase 2, mitochondrial	74.0	12.1	6.4	0.8	0.3	0.6	-0.79
radixin isoform 1	69.0	9.1	6.6	0.0	0.3	0.7	-1.00
delta-1-pyrroline-5-carboxylate dehydrogenase, mitochondrial	62.0	6.7	5.9	0.0	0.3	0.9	-1.00
60S ribosomal protein L11 isoform 1	20.0	8.5	1.9	0.0	0.3	0.2	-1.00
LETM1 and EF-hand domain-containing protein 1, mitochondrial	83.0	23.7	7.7	0.0	0.3	0.3	-1.00
tropomodulin-1 isoform 1	41.0	3.3	2.9	0.9	0.3	1.1	-0.55
transcription factor A, mitochondrial	29.0	2.7	2.7	0.0	0.3	1.0	-1.00
protein Niban	104.0	11.3	7.0	2.5	0.3	0.8	-0.47
clathrin heavy chain 1 isoform 1	192.0	16.4	14.3	3.1	0.3	1.1	-0.64
prostaglandin E synthase 2	42.0	14.3	3.7	0.0	0.2	0.3	-1.00
leucyl-cystinyl aminopeptidase	118.0	24.3	6.6	3.9	0.2	0.4	-0.26
cAMP-dependent protein kinase catalytic subunit beta isoform 1	46.0	5.3	3.2	0.9	0.2	0.8	-0.58
60S ribosomal protein L22 isoform 2	15.0	3.9	1.3	0.0	0.2	0.3	-1.00
protein NipSnap homolog 3A	28.0	5.9	2.5	0.0	0.2	0.4	-1.00
uncharacterized protein LOC476756 isoform 1	62.0	9.7	4.0	1.4	0.2	0.6	-0.48
glucose-6-phosphate isomerase isoform 1	63.0	7.0	4.4	0.8	0.2	0.8	-0.68
ribosomal protein SA	33.0	13.7	1.7	0.9	0.2	0.2	-0.34
long-chain specific acyl-CoA dehydrogenase, mitochondrial	49.0	3.8	3.8	0.0	0.2	1.0	-1.00
guanine nucleotide-binding protein G(i) subunit alpha-1	39.0	8.1	3.0	0.0	0.2	0.4	-1.00
septin-2	42.0	1.9	3.2	0.0	0.2	1.7	-1.00
plasma membrane calcium-transporting ATPase 4 isoform 3	129.0	11.3	4.3	5.5	0.2	0.9	0.12
T-complex protein 1 subunit alpha isoform 1	60.0	2.9	4.4	0.0	0.2	1.5	-1.00
40S ribosomal protein S18	18.0	2.6	1.3	0.0	0.2	0.5	-1.00

neuroblast differentiation-associated protein AHNAK	580.0	40.0	39.8	2.9	0.2	1.1	-0.86
reticulon-4-interacting protein 1, mitochondrial isoform 1	44.0	3.5	3.2	0.0	0.2	0.9	-1.00
lysosome membrane protein 2	54.0	2.3	3.0	0.8	0.2	1.7	-0.56
mitochondrial import inner membrane translocase subunit Tim23-like	22.0	6.0	1.6	0.0	0.2	0.3	-1.00
integrin-linked protein kinase isoform 1	51.0	2.4	2.6	1.0	0.2	1.5	-0.45
NADP-dependent malic enzyme, mitochondrial isoform 2	67.0	3.9	4.4	0.0	0.2	1.2	-1.00
dynamin-like 120 kDa protein, mitochondrial isoform 2	118.0	10.1	7.0	0.8	0.2	0.8	-0.78
pyruvate dehydrogenase protein X component, mitochondrial isoform 1	54.0	21.8	3.6	0.0	0.2	0.2	-1.00
catenin beta-1	86.0	4.0	3.9	1.7	0.2	1.4	-0.38
cytosolic 5'-nucleotidase 1A	40.0	3.8	1.7	0.9	0.2	0.7	-0.34
eukaryotic initiation factor 4A-II isoform 7	46.0	6.7	2.9	0.0	0.2	0.4	-1.00
methylmalonyl-CoA mutase, mitochondrial isoform 2	83.0	6.1	5.2	0.0	0.2	0.9	-1.00
60S ribosomal protein L9	22.0	6.8	1.3	0.0	0.2	0.2	-1.00
EH domain-containing protein 1	62.0	9.8	3.7	0.0	0.2	0.4	-1.00
metaxin-1 isoform 2	50.0	3.5	3.0	0.0	0.2	0.9	-1.00
ferrochelatase, mitochondrial	45.0	2.9	1.7	0.9	0.2	0.9	-0.34
mitochondrial chaperone BCS1 isoform 1	47.0	13.9	2.6	0.0	0.2	0.2	-1.00
myosin-4	223.0	76.5	4.1	8.2	0.2	0.2	0.33
haptoglobin isoform 2	38.0	2.3	2.0	0.0	0.1	0.9	-1.00
serum deprivation-response protein	59.0	5.8	2.2	0.8	0.1	0.5	-0.45
2-oxoisovalerate dehydrogenase subunit beta, mitochondrial	42.0	2.5	2.1	0.0	0.1	0.9	-1.00
[Pyruvate dehydrogenase [lipoamide]] kinase isozyme 2, mitochondrial	46.0	1.2	1.5	0.9	0.1	2.0	-0.26
60S ribosomal protein L14	23.0	4.5	1.2	0.0	0.1	0.3	-1.00
neural cell adhesion molecule 1 precursor	80.0	4.3	1.6	2.5	0.1	0.9	0.22
T-complex protein 1 subunit theta isoform 1	60.0	3.9	3.0	0.0	0.1	0.8	-1.00
membrane primary amine oxidase-like	84.0	5.3	4.1	0.0	0.1	0.8	-1.00
myosin-9	226.0	10.8	10.9	0.0	0.1	1.0	-1.00
protein cordon-bleu	148.0	5.7	4.2	2.9	0.1	1.3	-0.18
ATP-binding cassette sub-family B member 8, mitochondrial	78.0	8.8	3.7	0.0	0.1	0.4	-1.00
nascent polypeptide-associated complex subunit alpha	23.0	13.1	1.1	0.0	0.1	0.1	-1.00
uncharacterized protein LOC478967	47.0	3.7	2.1	0.0	0.1	0.6	-1.00
beta-2-glycoprotein 1 precursor	38.0	5.6	1.7	0.0	0.1	0.3	-1.00
bcl-2-like protein 13	51.0	9.9	2.3	0.0	0.1	0.2	-1.00
mitofusin-2 isoform 10	86.0	4.6	3.0	0.8	0.1	0.8	-0.58
heterogeneous nuclear ribonucleoprotein K	51.0	3.0	2.2	0.0	0.1	0.7	-1.00
PDZ and LIM domain protein 1	55.0	5.0	2.3	0.0	0.1	0.5	-1.00
fermitin family homolog 2	71.0	2.3	3.0	0.0	0.1	1.3	-1.00
histone H1.2-like	21.0	9.9	0.9	0.0	0.1	0.1	-1.00
calcium-binding mitochondrial carrier protein SCaMC-1	54.0	2.2	2.2	0.0	0.1	1.0	-1.00
myosin-3	222.0	71.9	0.0	8.9	0.1	0.1	1.00
synaptic vesicle membrane protein VAT-1 homolog	43.0	20.0	1.7	0.0	0.1	0.1	-1.00
kidney mitochondrial carrier protein 1	32.0	6.0	1.2	0.0	0.1	0.2	-1.00
glycogen [starch] synthase, muscle	84.0	14.8	1.5	1.7	0.1	0.2	0.05
blood vessel epicardial substance	42.0	2.9	0.7	0.8	0.1	0.5	0.07
integrin alpha-5	143.0	3.4	3.4	1.6	0.1	1.4	-0.38
armadillo repeat-containing protein 1 isoform 1	31.0	2.3	1.1	0.0	0.1	0.5	-1.00
adenylate cyclase type 6	130.0	4.3	2.0	2.3	0.1	1.0	0.08
lipoamide acyltransferase component of branched-chain alpha-keto acid D	53.0	3.6	1.7	0.0	0.1	0.5	-1.00
transmembrane protein 143	53.0	8.4	1.7	0.0	0.1	0.2	-1.00
mitochondrial import receptor subunit TOM70	67.0	5.6	1.4	0.8	0.1	0.4	-0.31
40S ribosomal protein S2-like isoform 2	32.0	7.1	1.0	0.0	0.1	0.1	-1.00
serpin peptidase inhibitor, clade A member 1 precursor	46.0	1.0	1.5	0.0	0.1	1.5	-1.00
calcium-transporting ATPase type 2C member 1 isoform 10	104.0	3.3	0.8	2.5	0.1	1.0	0.53
hexokinase-1	103.0	98.9	3.2	0.0	0.1	0.0	-1.00
40S ribosomal protein S4, X isoform	30.0	9.2	0.9	0.0	0.1	0.1	-1.00
vesicle-fusing ATPase	72.0	6.8	2.2	0.0	0.1	0.3	-1.00
glutaryl-CoA dehydrogenase, mitochondrial	48.0	4.0	1.5	0.0	0.1	0.4	-1.00
propionyl-CoA carboxylase beta chain, mitochondrial isoform 1	58.0	1.9	1.8	0.0	0.1	0.9	-1.00
V-type proton ATPase subunit B, brain isoform isoform 1	57.0	3.1	0.9	0.8	0.1	0.6	-0.02
ankyrin-3 isoform 1	480.0	10.1	9.0	5.4	0.1	1.4	-0.25

60S ribosomal protein L6 isoform 1	33.0	3.5	1.0	0.0	0.1	0.3	-1.00
alpha-aminoadipic semialdehyde dehydrogenase	59.0	1.0	1.7	0.0	0.1	1.7	-1.00
vitronectin isoform 2	54.0	1.9	0.7	0.8	0.1	0.8	0.06
transferrin receptor protein 1	87.0	17.9	2.5	0.0	0.1	0.1	-1.00
methylcrotonoyl-CoA carboxylase beta chain, mitochondrial	62.0	1.9	1.8	0.0	0.1	0.9	-1.00
solute carrier family 44, member 2	80.0	1.5	2.3	0.0	0.1	1.5	-1.00
kinesin-1 heavy chain-like	110.0	2.7	3.1	0.0	0.1	1.2	-1.00
glycerol kinase	61.0	7.9	0.9	0.8	0.1	0.2	-0.02
protein-glutamine gamma-glutamyltransferase 2	78.0	6.8	2.2	0.0	0.1	0.3	-1.00
platelet endothelial cell adhesion molecule	83.0	2.6	2.3	0.0	0.1	0.9	-1.00
glycerol-3-phosphate dehydrogenase, mitochondrial isoform 2	78.0	8.3	2.2	0.0	0.1	0.3	-1.00
surfeit locus protein 1	34.0	3.6	0.9	0.0	0.1	0.3	-1.00
glycogenin-1	39.0	10.0	1.1	0.0	0.1	0.1	-1.00
perilipin-4 isoform 1	137.0	6.2	3.6	0.0	0.1	0.6	-1.00
erythrocyte membrane protein band 4.1-like 3 isoform 1	121.0	9.5	2.3	0.8	0.1	0.3	-0.47
podocalyxin precursor	60.0	1.7	1.6	0.0	0.1	0.9	-1.00
V-type proton ATPase 116 kDa subunit a isoform 1 isoform 1	96.0	7.0	2.4	0.0	0.1	0.3	-1.00
myosin-binding protein C, cardiac-type	141.0	134.5	2.0	1.6	0.1	0.0	-0.12
phosphoglucomutase-1 isoform 1	64.0	6.7	1.5	0.0	0.1	0.2	-1.00
mitochondrial import inner membrane translocase subunit TIM50	48.0	3.6	1.1	0.0	0.1	0.3	-1.00
decorin precursor	40.0	13.5	0.8	0.0	0.1	0.1	-1.00
mitochondrial Rho GTPase 1	77.0	6.1	1.5	0.0	0.1	0.3	-1.00
leucine-rich PPR motif-containing protein, mitochondrial	158.0	9.5	3.1	0.0	0.1	0.3	-1.00
dynactin subunit 2 isoform 3	44.0	2.4	0.9	0.0	0.1	0.4	-1.00
propionyl-CoA carboxylase alpha chain, mitochondrial isoform 2	81.0	3.0	1.5	0.0	0.1	0.5	-1.00
elongation factor 2	95.0	10.0	1.7	0.0	0.0	0.2	-1.00
lysosome-associated membrane glycoprotein 2 isoform 4	45.0	3.2	0.8	0.0	0.0	0.2	-1.00
ATP-binding cassette sub-family B member 7, mitochondrial	83.0	2.0	1.4	0.0	0.0	0.7	-1.00
l-2-hydroxyglutarate dehydrogenase, mitochondrial isoform 2	51.0	2.9	0.9	0.0	0.0	0.3	-1.00
vacuolar protein sorting-associated protein 35 isoform 1	92.0	6.8	1.5	0.0	0.0	0.2	-1.00
tRNA-splicing ligase RtcB homolog	55.0	4.9	0.0	0.9	0.0	0.2	1.00
importin subunit beta-1 isoform 1	97.0	1.6	1.5	0.0	0.0	1.0	-1.00
acylglycerol kinase, mitochondrial	47.0	2.2	0.7	0.0	0.0	0.3	-1.00
isoleucyl-tRNA synthetase, mitochondrial, partial	108.0	10.4	1.7	0.0	0.0	0.2	-1.00
uncharacterized protein LOC478914	200.0	4.6	3.1	0.0	0.0	0.7	-1.00
UTP--glucose-1-phosphate uridylyltransferase isoform 2	56.0	3.4	0.9	0.0	0.0	0.3	-1.00
myomesin-1 isoform 2	188.0	13.9	1.3	1.6	0.0	0.2	0.08
tyrosine-protein phosphatase non-receptor type substrate 1	55.0	2.5	0.8	0.0	0.0	0.3	-1.00
neuropilin-1 isoform 2	103.0	0.9	1.6	0.0	0.0	1.8	-1.00
2-oxoisovalerate dehydrogenase subunit alpha, mitochondrial isoform 2	51.0	5.4	0.7	0.0	0.0	0.1	-1.00
Niemann-Pick C1 protein precursor	142.0	1.9	2.0	0.0	0.0	1.1	-1.00
prostaglandin F2 receptor negative regulator	112.0	0.9	1.6	0.0	0.0	1.8	-1.00
mitochondrial antiviral-signaling protein	55.0	4.6	0.7	0.0	0.0	0.2	-1.00
SPEG complex locus	354.0	17.0	3.5	0.7	0.0	0.2	-0.65
cytoplasmic dynein 1 heavy chain 1 isoform 1	532.0	31.7	6.1	0.0	0.0	0.2	-1.00
collagen alpha-6(VI) chain	248.0	7.6	2.0	0.7	0.0	0.4	-0.46
myomesin-2, partial	121.0	12.9	1.3	0.0	0.0	0.1	-1.00
syntaxin-binding protein 3 isoform 1	68.0	3.0	0.7	0.0	0.0	0.2	-1.00
catenin alpha-1	100.0	4.2	0.0	0.9	0.0	0.2	1.00
AFG3-like protein 2	90.0	16.7	0.8	0.0	0.0	0.0	-1.00
oxysterol-binding protein 1	91.0	1.7	0.8	0.0	0.0	0.5	-1.00
AP-2 complex subunit alpha-2	106.0	1.7	0.8	0.0	0.0	0.5	-1.00
NLR family member X1	107.0	16.7	0.8	0.0	0.0	0.0	-1.00
huntingtin-interacting protein 1	116.0	1.7	0.8	0.0	0.0	0.5	-1.00
obscurin	815.0	6.2	1.1	2.0	0.0	0.5	0.29
cardiomyopathy-associated protein 5	441.0	1.1	1.5	0.0	0.0	1.4	-1.00
heterogeneous nuclear ribonucleoproteins A2/B1 isoform 1	37.0	19.1	0.0	0.0	0.0	0.0	N/A
troponin T type 2, cardiac	34.0	12.3	0.0	0.0	0.0	0.0	N/A
optic atrophy 3 protein	21.0	6.1	0.0	0.0	0.0	0.0	N/A
transmembrane protein 126A	22.0	6.0	0.0	0.0	0.0	0.0	N/A

	myosin, heavy chain 13, skeletal muscle	224.0	60.3	0.0	0.0	0.0	0.0	N/A
	60S ribosomal protein L26	17.0	4.5	0.0	0.0	0.0	0.0	N/A
	UPF0723 protein C11orf83 homolog	10.0	2.6	0.0	0.0	0.0	0.0	N/A
	heterogeneous nuclear ribonucleoprotein C-like 1	32.0	8.0	0.0	0.0	0.0	0.0	N/A
	DNA-binding protein A	32.0	6.7	0.0	0.0	0.0	0.0	N/A
	60S ribosomal protein L30 isoform 1	13.0	2.6	0.0	0.0	0.0	0.0	N/A
	ELAV-like protein 1-like	36.0	7.0	0.0	0.0	0.0	0.0	N/A
	protein CCSMST1	14.0	2.6	0.0	0.0	0.0	0.0	N/A
	heat shock-related 70 kDa protein 2 isoform 1	70.0	11.4	0.0	0.0	0.0	0.0	N/A
	heterogeneous nuclear ribonucleoprotein D0 isoform 2	33.0	5.4	0.0	0.0	0.0	0.0	N/A
	40S ribosomal protein S19	16.0	2.6	0.0	0.0	0.0	0.0	N/A
	UPF0459 protein C19orf50 homolog isoform 1	20.0	3.2	0.0	0.0	0.0	0.0	N/A
	guanine nucleotide binding protein (G protein), beta polypeptide 2-like 1 isc	30.0	4.7	0.0	0.0	0.0	0.0	N/A
	dual specificity protein phosphatase 3	26.0	4.1	0.0	0.0	0.0	0.0	N/A
	microtubule-associated protein 4 isoform 1	120.0	18.5	0.0	0.0	0.0	0.0	N/A
	hexokinase-2-like	101.0	14.9	0.0	0.0	0.0	0.0	N/A
	coiled-coil domain-containing protein 90B, mitochondrial isoform 1	29.0	4.1	0.0	0.0	0.0	0.0	N/A
	retinol dehydrogenase 13	36.0	5.0	0.0	0.0	0.0	0.0	N/A
	40S ribosomal protein S10	19.0	2.6	0.0	0.0	0.0	0.0	N/A
	copine-3	60.0	8.0	0.0	0.0	0.0	0.0	N/A
	40S ribosomal protein S16	20.0	2.6	0.0	0.0	0.0	0.0	N/A
	uncharacterized protein LOC478881 isoform 1	25.0	3.2	0.0	0.0	0.0	0.0	N/A
	ATPase family AAA domain-containing protein 3-like	67.0	8.1	0.0	0.0	0.0	0.0	N/A
	vacuolar protein sorting-associated protein 26A isoform 1	39.0	4.6	0.0	0.0	0.0	0.0	N/A
	40S ribosomal protein S3a isoform 1	30.0	3.6	0.0	0.0	0.0	0.0	N/A
	heterogeneous nuclear ribonucleoprotein H3 isoform 3	35.0	3.7	0.0	0.0	0.0	0.0	N/A
	probable ATP-dependent RNA helicase DDX17 isoform 11	73.0	6.9	0.0	0.0	0.0	0.0	N/A
	major vault protein	99.0	9.2	0.0	0.0	0.0	0.0	N/A
	heterogeneous nuclear ribonucleoprotein G isoform 6	42.0	3.9	0.0	0.0	0.0	0.0	N/A
	heterogeneous nuclear ribonucleoprotein M isoform 2	79.0	7.1	0.0	0.0	0.0	0.0	N/A
	calpastatin isoform 3	81.0	6.9	0.0	0.0	0.0	0.0	N/A
	ras GTPase-activating protein-binding protein 1 isoform 4	52.0	4.0	0.0	0.0	0.0	0.0	N/A
	phosphoglucomutase 5	55.0	3.9	0.0	0.0	0.0	0.0	N/A
	prolargin	43.0	3.0	0.0	0.0	0.0	0.0	N/A
	secretory carrier-associated membrane protein 2 isoform 1	37.0	2.5	0.0	0.0	0.0	0.0	N/A
	polyadenylate-binding protein 4 isoform 2	71.0	4.8	0.0	0.0	0.0	0.0	N/A
	solute carrier family 25 member 40	38.0	2.5	0.0	0.0	0.0	0.0	N/A
	28S ribosomal protein S29, mitochondrial isoform 2	41.0	2.3	0.0	0.0	0.0	0.0	N/A
	protein transport protein Sec23A isoform 2	86.0	4.6	0.0	0.0	0.0	0.0	N/A
	choline dehydrogenase, mitochondrial	76.0	3.9	0.0	0.0	0.0	0.0	N/A
	AP-1 complex subunit beta-1 isoform 3	106.0	5.3	0.0	0.0	0.0	0.0	N/A
	T-complex protein 1 subunit delta-like	58.0	2.9	0.0	0.0	0.0	0.0	N/A
	spliceosome RNA helicase DDX39B	49.0	2.4	0.0	0.0	0.0	0.0	N/A
	ATP-dependent RNA helicase DDX1 isoform 2	82.0	3.9	0.0	0.0	0.0	0.0	N/A
	heterogeneous nuclear ribonucleoprotein U isoform 1	96.0	4.5	0.0	0.0	0.0	0.0	N/A
	phenylalanyl-tRNA synthetase beta chain isoform 1	66.0	3.0	0.0	0.0	0.0	0.0	N/A
	dihydroorotate dehydrogenase (quinone), mitochondrial	43.0	1.9	0.0	0.0	0.0	0.0	N/A
	rab GDP dissociation inhibitor beta	50.0	2.2	0.0	0.0	0.0	0.0	N/A
	elongation factor 1-gamma isoform 1	50.0	2.2	0.0	0.0	0.0	0.0	N/A
	collagen alpha-1(XV) chain	140.0	5.4	0.0	0.0	0.0	0.0	N/A
	ATP-binding cassette sub-family B member 10, mitochondrial	75.0	2.9	0.0	0.0	0.0	0.0	N/A
	mitochondrial import inner membrane translocase subunit TIM44	52.0	1.9	0.0	0.0	0.0	0.0	N/A
	lactamase, beta	53.0	1.9	0.0	0.0	0.0	0.0	N/A
	T-complex protein 1 subunit beta isoform 1	57.0	2.1	0.0	0.0	0.0	0.0	N/A
	non-POU domain-containing octamer-binding protein isoform 2	54.0	1.9	0.0	0.0	0.0	0.0	N/A
	vigilin isoform 3	146.0	5.2	0.0	0.0	0.0	0.0	N/A
	splicing factor, proline- and glutamine-rich	70.0	2.5	0.0	0.0	0.0	0.0	N/A
	atlastin-3	59.0	2.1	0.0	0.0	0.0	0.0	N/A
	putative hexokinase HKDC1	103.0	3.6	0.0	0.0	0.0	0.0	N/A
	staphylococcal nuclease domain-containing protein 1	102.0	3.6	0.0	0.0	0.0	0.0	N/A

pentatricopeptide repeat-containing protein 3, mitochondrial	78.0	2.3	0.0	0.0	0.0	0.0	N/A
proline dehydrogenase 1, mitochondrial	72.0	2.0	0.0	0.0	0.0	0.0	N/A
uncharacterized protein LOC100683193	57.0	1.5	0.0	0.0	0.0	0.0	N/A
dynamin-2	98.0	2.5	0.0	0.0	0.0	0.0	N/A
coatomer subunit gamma	98.0	2.5	0.0	0.0	0.0	0.0	N/A
caseinolytic peptidase B protein homolog isoform 3	80.0	1.9	0.0	0.0	0.0	0.0	N/A
ATP-dependent zinc metalloprotease YME1L1 isoform 2	80.0	1.9	0.0	0.0	0.0	0.0	N/A
erythrocyte membrane protein band 4.1-like 2 isoform 2	109.0	2.6	0.0	0.0	0.0	0.0	N/A
cytoplasmic dynein 1 intermediate chain 2 isoform 3	68.0	1.5	0.0	0.0	0.0	0.0	N/A
protein KIAA0664 isoform 1	166.0	3.5	0.0	0.0	0.0	0.0	N/A
c-1-tetrahydrofolate synthase, cytoplasmic	101.0	1.8	0.0	0.0	0.0	0.0	N/A
protein transport protein Sec24C-like isoform 3	107.0	1.7	0.0	0.0	0.0	0.0	N/A
rho guanine nucleotide exchange factor 10	170.0	2.6	0.0	0.0	0.0	0.0	N/A
importin-5 isoform 1	121.0	1.8	0.0	0.0	0.0	0.0	N/A
anion exchange protein 3 isoform 1	135.0	1.7	0.0	0.0	0.0	0.0	N/A
chondroitin sulfate proteoglycan 4	245.0	2.2	0.0	0.0	0.0	0.0	N/A
**253 proteins not detected in starting MVs - in order of decreasing ASR**							
3-ketoacyl-CoA thiolase, peroxisomal isoform 1	44.0	0.0	77.3	46.5	7.8	N/A	-0.25
peroxisomal membrane protein 11B isoform 1	28.0	0.0	56.7	8.7	6.5	N/A	-0.73
phytanoyl-CoA hydroxylase-like	39.0	0.0	72.9	17.3	6.4	N/A	-0.62
non-specific lipid-transfer protein isoform 3	58.0	0.0	67.0	32.1	4.8	N/A	-0.35
lactamase, beta 2 isoform 1	33.0	0.0	40.1	9.1	4.2	N/A	-0.63
phytanoyl-CoA hydroxylase-like	39.0	0.0	40.6	9.3	3.6	N/A	-0.63
enoyl-CoA delta isomerase 2, mitochondrial	41.0	0.0	37.9	9.6	3.2	N/A	-0.60
immediate early response 3-interacting protein 1	9.0	0.0	2.7	6.4	2.8	N/A	0.41
D-aspartate oxidase	38.0	0.0	26.3	10.9	2.7	N/A	-0.42
tubulin beta chain	42.0	0.0	19.3	21.6	2.7	N/A	0.06
peroxisomal acyl-coenzyme A oxidase 1 isoform 1	75.0	0.0	56.6	15.8	2.7	N/A	-0.56
calumenin isoform 2	37.0	0.0	10.3	24.6	2.6	N/A	0.41
neudesin	15.0	0.0	1.3	12.6	2.6	N/A	0.81
receptor expression-enhancing protein 5-like	12.0	0.0	4.0	7.0	2.6	N/A	0.28
signal peptidase complex catalytic subunit SEC11A	21.0	0.0	9.3	9.8	2.5	N/A	0.03
glutathione peroxidase 3 precursor	25.0	0.0	8.0	13.4	2.4	N/A	0.25
uncharacterized protein C4orf32-like	15.0	0.0	5.7	6.9	2.3	N/A	0.10
peptidyl-tRNA hydrolase 2, mitochondrial isoform 1	19.0	0.0	7.4	7.8	2.2	N/A	0.03
glycophorin-C	8.0	0.0	6.3	0.0	2.2	N/A	-1.00
transmembrane emp24 domain-containing protein 4 isoform 1	25.0	0.0	9.5	10.2	2.2	N/A	0.04
3-ketodihydrosphingosine reductase	48.0	0.0	19.2	17.6	2.1	N/A	-0.04
tubulin alpha-4A chain isoform 1	50.0	0.0	19.0	18.7	2.1	N/A	-0.01
transmembrane protein 85	20.0	0.0	10.2	4.2	2.0	N/A	-0.42
transmembrane protein 199	23.0	0.0	6.9	9.6	2.0	N/A	0.16
signal peptidase complex subunit 3	20.0	0.0	6.3	7.2	1.9	N/A	0.07
protein canopy homolog 2 isoform 1	21.0	0.0	8.5	5.6	1.9	N/A	-0.21
reticulon-3	25.0	0.0	6.3	10.3	1.9	N/A	0.24
transmembrane emp24 domain-containing protein 7 precursor	25.0	0.0	4.7	11.8	1.8	N/A	0.43
vesicle transport protein USE1	29.0	0.0	6.5	12.4	1.8	N/A	0.31
UPF0556 protein C19orf10 homolog	19.0	0.0	4.0	8.4	1.8	N/A	0.36
acyl-coenzyme A thioesterase 8 isoform 1	36.0	0.0	17.9	5.5	1.8	N/A	-0.53
peroxisomal acyl-coenzyme A oxidase 3	79.0	0.0	37.8	11.5	1.7	N/A	-0.53
dolichol-phosphate mannosyltransferase isoform 6	29.0	0.0	7.5	10.1	1.7	N/A	0.15
thioredoxin-related transmembrane protein 1	32.0	0.0	9.0	9.8	1.6	N/A	0.04
2-acylglycerol O-acyltransferase 1	39.0	0.0	2.9	19.6	1.6	N/A	0.74
peroxisomal membrane protein 4	24.0	0.0	13.8	0.0	1.6	N/A	-1.00
peroxisomal membrane protein PEX14	46.0	0.0	22.5	2.5	1.5	N/A	-0.80
selenoprotein T precursor	22.0	0.0	4.0	7.0	1.4	N/A	0.28
Golgi SNAP receptor complex member 2	25.0	0.0	2.5	9.9	1.4	N/A	0.60
derlin-1 isoform 2	29.0	0.0	5.3	9.0	1.4	N/A	0.26
mesencephalic astrocyte-derived neurotrophic factor-like	20.0	0.0	4.0	5.6	1.3	N/A	0.17
myosin regulatory light polypeptide 9-like isoform 1	20.0	0.0	9.4	0.0	1.3	N/A	-1.00
glycerol-3-phosphate acyltransferase 3	48.0	0.0	5.4	16.7	1.3	N/A	0.51

	peroxisomal membrane protein 11A-like	28.0	0.0	11.4	1.4	1.3	N/A	-0.78
	ras-related protein Rab-4A	24.0	0.0	3.5	7.5	1.3	N/A	0.36
	leucine-rich repeat-containing protein 59	35.0	0.0	7.0	9.0	1.3	N/A	0.13
	UPF0510 protein INM02 isoform 1	27.0	0.0	5.7	5.5	1.2	N/A	-0.02
	probable lipid phosphate phosphatase PPAPDC3	30.0	0.0	3.6	8.7	1.1	N/A	0.42
	monoacylglycerol lipase ABHD6	38.0	0.0	4.3	10.9	1.1	N/A	0.44
	dihydroxyacetone phosphate acyltransferase	78.0	0.0	22.2	7.9	1.1	N/A	-0.47
	peroxisomal membrane protein PEX16 isoform 1	39.0	0.0	12.8	2.1	1.1	N/A	-0.72
	ribonuclease UK114 isoform 1	14.0	0.0	5.3	0.0	1.1	N/A	-1.00
	3 beta-hydroxysteroid dehydrogenase type 7	41.0	0.0	6.4	8.7	1.0	N/A	0.15
	barrier-to-autointegration factor isoform 1	20.0	0.0	1.3	5.6	1.0	N/A	0.62
	2-hydroxyacyl-CoA lyase 1	64.0	0.0	19.4	1.7	0.9	N/A	-0.84
	lactotransferrin isoform 1	77.0	0.0	24.3	0.8	0.9	N/A	-0.93
	peroxisomal biogenesis factor 3	42.0	0.0	7.4	5.7	0.9	N/A	-0.13
	RAB, member of RAS oncogene family-like 3	26.0	0.0	3.7	4.3	0.9	N/A	0.07
	endoplasmic reticulum-Golgi intermediate compartment protein 1 isoform	33.0	0.0	3.1	6.8	0.8	N/A	0.38
	ethanolaminephosphotransferase 1	45.0	0.0	3.7	9.4	0.8	N/A	0.43
	transmembrane 6 superfamily member 1 isoform 2	42.0	0.0	5.3	6.9	0.8	N/A	0.13
	UPF0670 protein C8orf55 homolog	17.0	0.0	3.1	1.8	0.8	N/A	-0.27
	arylacetamide deacetylase	46.0	0.0	4.4	8.9	0.8	N/A	0.33
	syntaxin-8 isoform 1	27.0	0.0	2.7	5.1	0.8	N/A	0.31
	torsin-1B-like	38.0	0.0	2.2	8.6	0.8	N/A	0.59
	protein YIF1B isoform 1	34.0	0.0	2.9	6.7	0.8	N/A	0.40
	cofilin-1 isoform 1	19.0	0.0	5.3	0.0	0.8	N/A	-1.00
	protein phosphatase 1L	41.0	0.0	3.4	7.4	0.7	N/A	0.36
	dehydrogenase/reductase SDR family member on chromosome X	42.0	0.0	4.1	6.6	0.7	N/A	0.23
	GPI-anchor transamidase isoform 2	46.0	0.0	1.7	10.0	0.7	N/A	0.70
	GTPase HRas isoform 2	21.0	0.0	5.3	0.0	0.7	N/A	-1.00
	peroxisomal carnitine O-octanoyltransferase isoform 1	70.0	0.0	14.7	2.5	0.7	N/A	-0.71
	gap junction alpha-1 protein	43.0	0.0	8.6	2.0	0.7	N/A	-0.63
	transmembrane protein 56-like	30.0	0.0	3.2	4.1	0.7	N/A	0.12
	heat shock protein beta-8	22.0	0.0	2.3	3.0	0.7	N/A	0.13
	neutrophil elastase precursor	30.0	0.0	7.1	0.0	0.7	N/A	-1.00
	ERO1-like protein beta	53.0	0.0	4.4	8.0	0.7	N/A	0.28
	lysophospholipid acyltransferase 7 isoform 1	53.0	0.0	4.4	7.6	0.6	N/A	0.27
	syntaxin-18 isoform 1	39.0	0.0	2.6	6.1	0.6	N/A	0.40
	thioredoxin domain-containing protein 12	19.0	0.0	0.0	4.2	0.6	N/A	1.00
	acyl-CoA-binding domain-containing protein 4 isoform 1	34.0	0.0	7.4	0.0	0.6	N/A	-1.00
	fat storage-inducing transmembrane protein 1	32.0	0.0	2.5	4.5	0.6	N/A	0.29
	transmembrane emp24 domain-containing protein 5 isoform 2	26.0	0.0	1.2	4.5	0.6	N/A	0.59
	peroxiredoxin-5, mitochondrial	37.0	0.0	6.6	1.4	0.6	N/A	-0.65
	peroxisome biogenesis factor 2	35.0	0.0	5.8	1.4	0.6	N/A	-0.60
	erlin-1	39.0	0.0	2.6	5.4	0.6	N/A	0.35
	prolactin regulatory element-binding protein isoform 1	46.0	0.0	2.9	6.3	0.6	N/A	0.37
	glycerol-3-phosphate acyltransferase 4	52.0	0.0	1.7	8.5	0.5	N/A	0.66
	uncharacterized protein LOC609292	19.0	0.0	1.7	2.0	0.5	N/A	0.06
	dolichyl-diphosphooligosaccharide--protein glycosyltransferase subunit ST	81.0	0.0	4.7	11.0	0.5	N/A	0.40
	uncharacterized protein LOC478822	150.0	0.0	13.4	15.7	0.5	N/A	0.08
	purine nucleoside phosphorylase isoform 1	32.0	0.0	6.2	0.0	0.5	N/A	-1.00
	glucose-6-phosphate translocase isoform 7	46.0	0.0	2.0	6.9	0.5	N/A	0.54
	Golgi SNAP receptor complex member 1	29.0	0.0	1.2	4.3	0.5	N/A	0.55
	dolichyl-phosphate beta-glucosyltransferase isoform 6	34.0	0.0	2.1	4.3	0.5	N/A	0.33
	glycosylphosphatidylinositol anchor attachment 1 protein	68.0	0.0	5.9	6.6	0.5	N/A	0.06
	isochorismatase domain-containing protein 2, mitochondrial isoform 1	22.0	0.0	4.0	0.0	0.5	N/A	-1.00
	dnaJ homolog subfamily B member 6 isoform 2	36.0	0.0	3.3	3.3	0.5	N/A	0.00
	protein CYR61	42.0	0.0	4.3	3.3	0.5	N/A	-0.13
	tropomyosin alpha-3 chain	29.0	0.0	5.2	0.0	0.5	N/A	-1.00
	protein FAM134B	49.0	0.0	2.4	6.1	0.5	N/A	0.43
	RAB21, member RAS oncogene family, partial	24.0	0.0	1.2	3.0	0.5	N/A	0.44
	ankyrin repeat domain-containing protein 46 isoform 1	25.0	0.0	1.3	3.0	0.5	N/A	0.38

	etoposide-induced protein 2.4 homolog isoform 1	39.0	0.0	2.8	3.9	0.5	N/A	0.17
	dnaJ homolog subfamily B member 12 isoform 7	42.0	0.0	1.1	6.1	0.5	N/A	0.71
	N-acetylglucosamine-6-sulfatase	53.0	0.0	3.1	5.9	0.5	N/A	0.31
	thioredoxin domain-containing protein 5	55.0	0.0	1.7	7.6	0.5	N/A	0.63
	peripherin isoform 1	53.0	0.0	1.8	7.2	0.5	N/A	0.60
	actin-related protein 2/3 complex subunit 5	16.0	0.0	2.7	0.0	0.5	N/A	-1.00
	reticulocalbin-1	37.0	0.0	0.0	6.0	0.5	N/A	1.00
	microfibrillar-associated protein 5 isoform 1	18.0	0.0	0.0	2.9	0.4	N/A	1.00
	chloride intracellular channel protein 1	27.0	0.0	3.1	1.2	0.4	N/A	-0.45
	ADP-ribosylation factor-like 1 isoform 1	20.0	0.0	3.1	0.0	0.4	N/A	-1.00
	peroxisome assembly protein 26	34.0	0.0	5.3	0.0	0.4	N/A	-1.00
	calcium-independent phospholipase A2-gamma isoform 1	88.0	0.0	12.2	1.5	0.4	N/A	-0.78
	retinol dehydrogenase 11	35.0	0.0	1.9	3.5	0.4	N/A	0.30
	dystroglycan precursor	97.0	0.0	5.8	8.5	0.4	N/A	0.19
	maleylacetoacetate isomerase isoform 2	24.0	0.0	3.5	0.0	0.4	N/A	-1.00
	apolipoprotein A-IV	44.0	0.0	2.6	3.7	0.4	N/A	0.17
	coenzyme Q-binding protein COQ10 homolog B, mitochondrial	27.0	0.0	3.8	0.0	0.4	N/A	-1.00
	calmegin	70.0	0.0	4.6	5.1	0.4	N/A	0.05
	transmembrane protein C9orf5	97.0	0.0	5.5	7.7	0.4	N/A	0.17
	N(4)-(beta-N-acetylglucosaminyl)-L-asparaginase isoform 1	37.0	0.0	5.0	0.0	0.4	N/A	-1.00
	metallo-beta-lactamase domain-containing protein 2	31.0	0.0	1.9	2.3	0.4	N/A	0.11
	cerebral dopamine neurotrophic factor	21.0	0.0	0.0	2.8	0.4	N/A	1.00
	uncharacterized protein C6orf47	33.0	0.0	2.3	2.1	0.4	N/A	-0.04
	cysteine-rich with EGF-like domains 1	46.0	0.0	3.0	2.8	0.4	N/A	-0.03
	lon protease homolog 2, peroxisomal-like isoform 1	95.0	0.0	11.7	0.0	0.3	N/A	-1.00
	transgelin-2	22.0	0.0	2.7	0.0	0.3	N/A	-1.00
	DDRGK domain-containing protein 1	36.0	0.0	1.5	2.8	0.3	N/A	0.32
	acyl-coenzyme A thioesterase 4	46.0	0.0	3.5	2.0	0.3	N/A	-0.28
	diablo homolog, mitochondrial	27.0	0.0	3.1	0.0	0.3	N/A	-1.00
	dnaJ homolog subfamily C member 10 isoform 2	91.0	0.0	4.6	5.9	0.3	N/A	0.12
	fibulin-5	50.0	0.0	0.9	4.8	0.3	N/A	0.70
	transmembrane protein 120B	40.0	0.0	0.9	3.5	0.3	N/A	0.58
	ancient ubiquitous protein 1 isoform 2	46.0	0.0	0.7	4.3	0.3	N/A	0.71
	V-type proton ATPase subunit S1 isoform 1	52.0	0.0	0.7	5.0	0.3	N/A	0.75
	citrate lyase beta like	38.0	0.0	4.1	0.0	0.3	N/A	-1.00
	dnaJ homolog subfamily C member 3	56.0	0.0	0.9	5.2	0.3	N/A	0.71
	rho GDP-dissociation inhibitor 1 isoform 2	23.0	0.0	2.5	0.0	0.3	N/A	-1.00
	protein FAM3C	25.0	0.0	2.7	0.0	0.3	N/A	-1.00
	nucleoside diphosphate-linked moiety X motif 19, mitochondrial	32.0	0.0	3.2	0.0	0.3	N/A	-1.00
	prostacyclin synthase-like	54.0	0.0	3.5	2.0	0.3	N/A	-0.28
	UPF0420 protein C16orf58 homolog	51.0	0.0	1.6	3.5	0.3	N/A	0.37
	peroxisome assembly protein 12 isoform 1	41.0	0.0	4.1	0.0	0.3	N/A	-1.00
	erythrocyte membrane protein band 4.2	77.0	0.0	5.9	1.6	0.3	N/A	-0.57
	grpE protein homolog 1, mitochondrial	24.0	0.0	2.3	0.0	0.3	N/A	-1.00
	chloride channel CLIC-like 1 isoform 1	62.0	0.0	2.9	2.8	0.3	N/A	-0.03
	sec1 family domain-containing protein 2 isoform 2	75.0	0.0	1.4	5.4	0.3	N/A	0.58
	diacylglycerol O-acyltransferase 1 isoform 1	56.0	0.0	0.7	4.3	0.3	N/A	0.71
	protein YIF1A isoform 2	32.0	0.0	0.0	2.9	0.3	N/A	1.00
	ceramide synthase 4	46.0	0.0	0.0	4.1	0.2	N/A	1.00
	starch-binding domain-containing protein 1	36.0	0.0	0.0	3.2	0.2	N/A	1.00
	acid ceramidase	49.0	0.0	3.3	1.1	0.2	N/A	-0.51
	biotinidase	62.0	0.0	3.0	2.5	0.2	N/A	-0.09
	sphingosine-1-phosphate lyase 1	63.0	0.0	1.8	3.6	0.2	N/A	0.34
	lamina-associated polypeptide 2, isoforms beta/delta/epsilon/gamma isof	51.0	0.0	0.0	4.3	0.2	N/A	1.00
	keratin, type I cuticular Ha3-II	47.0	0.0	1.3	2.6	0.2	N/A	0.32
	ubiquitin-associated domain-containing protein 2 isoform 2	39.0	0.0	1.0	2.3	0.2	N/A	0.39
	uncharacterized protein LOC475884	82.0	0.0	2.3	4.4	0.2	N/A	0.31
	ubiquitin carboxyl-terminal hydrolase 2 isoform 2	68.0	0.0	5.5	0.0	0.2	N/A	-1.00
	protein 4.1	91.0	0.0	7.2	0.0	0.2	N/A	-1.00
	lysophospholipid acyltransferase 2	60.0	0.0	1.9	2.8	0.2	N/A	0.19

platelet-activating factor acetylhydrolase 2, cytoplasmic	44.0	0.0	3.5	0.0	0.2	N/A	-1.00
enoyl-CoA hydratase domain-containing protein 2, mitochondrial	32.0	0.0	2.5	0.0	0.2	N/A	-1.00
enoyl-CoA hydratase domain-containing protein 3, mitochondrial	32.0	0.0	2.5	0.0	0.2	N/A	-1.00
tyrosine-protein phosphatase non-receptor type 1	49.0	0.0	1.7	2.0	0.2	N/A	0.06
fibrinogen gamma chain isoform 1	49.0	0.0	1.7	2.0	0.2	N/A	0.06
E3 ubiquitin-protein ligase MARCH5 isoform 1	31.0	0.0	2.3	0.0	0.2	N/A	-1.00
monoacylglycerol lipase ABHD12	45.0	0.0	0.7	2.6	0.2	N/A	0.56
centromere/kinetochore protein zw10 homolog isoform 1	89.0	0.0	2.3	4.2	0.2	N/A	0.29
acetyl-coenzyme A transporter 1 isoform 1	61.0	0.0	0.7	3.7	0.2	N/A	0.67
alpha-2-macroglobulin	165.0	0.0	4.0	7.8	0.2	N/A	0.32
transmembrane protein 135 isoform 1	52.0	0.0	3.7	0.0	0.2	N/A	-1.00
sialate O-acetylesterase isoform 2	57.0	0.0	1.5	2.5	0.2	N/A	0.26
minor histocompatibility antigen H13 isoform 2	47.0	0.0	0.0	3.3	0.2	N/A	1.00
omega-amidase NIT2	40.0	0.0	2.8	0.0	0.2	N/A	-1.00
reticulocalbin-2	37.0	0.0	0.9	1.7	0.2	N/A	0.33
heat shock 70 kDa protein 13	52.0	0.0	0.0	3.6	0.2	N/A	1.00
keratin, type II cuticular Hb1 isoform 1	56.0	0.0	1.3	2.5	0.2	N/A	0.30
fibrinogen alpha chain	97.0	0.0	3.9	2.6	0.2	N/A	-0.20
endoplasmic reticulum-Golgi intermediate compartment protein 3 isoform [illegible]	43.0	0.0	0.9	2.0	0.2	N/A	0.39
chloride intracellular channel protein 4	29.0	0.0	1.9	0.0	0.2	N/A	-1.00
actin-related protein 3 isoform 2	42.0	0.0	1.7	1.0	0.2	N/A	-0.28
myeloperoxidase	83.0	0.0	5.3	0.0	0.2	N/A	-1.00
carbonyl reductase [NADPH] 1	31.0	0.0	2.0	0.0	0.2	N/A	-1.00
cutaneous T-cell lymphoma-associated antigen 5 isoform 1	90.0	0.0	0.7	4.8	0.2	N/A	0.73
3-keto-steroid reductase	37.0	0.0	0.0	2.3	0.2	N/A	1.00
39S ribosomal protein L15, mitochondrial-like	34.0	0.0	2.1	0.0	0.2	N/A	-1.00
7-dehydrocholesterol reductase	54.0	0.0	1.1	2.2	0.2	N/A	0.35
lipase maturation factor 1	63.0	0.0	0.9	2.9	0.2	N/A	0.55
prenylcysteine oxidase 1 like	55.0	0.0	0.7	2.5	0.2	N/A	0.55
beta-1,3-glucosyltransferase	57.0	0.0	0.0	3.4	0.2	N/A	1.00
uncharacterized protein KIAA0564 isoform 3	217.0	0.0	12.5	0.0	0.2	N/A	-1.00
dimethylaniline monooxygenase [N-oxide-forming] 5 isoform 1	60.0	0.0	0.9	2.5	0.2	N/A	0.48
renin receptor-like	39.0	0.0	0.0	2.2	0.2	N/A	1.00
procollagen galactosyltransferase 1	72.0	0.0	0.0	4.0	0.2	N/A	1.00
regulator of microtubule dynamics protein 2 isoform 1	47.0	0.0	0.0	2.6	0.2	N/A	1.00
[Pyruvate dehydrogenase [lipoamide]] kinase isozyme 1, mitochondrial	42.0	0.0	2.2	0.0	0.1	N/A	-1.00
alpha-1,2-mannosyltransferase ALG9 isoform 2	70.0	0.0	0.9	2.7	0.1	N/A	0.52
ufm1-specific protease 2	56.0	0.0	0.9	2.0	0.1	N/A	0.39
actin-binding LIM protein 1	82.0	0.0	1.5	2.5	0.1	N/A	0.23
ornithine aminotransferase, mitochondrial isoform 2	48.0	0.0	2.3	0.0	0.1	N/A	-1.00
amyloid beta (A4) precursor-like protein 2 isoform 1	87.0	0.0	0.8	3.4	0.1	N/A	0.63
transmembrane and TPR repeat-containing protein 3	104.0	0.0	1.5	3.4	0.1	N/A	0.38
peroxisome assembly factor 2 isoform 1	104.0	0.0	5.0	0.0	0.1	N/A	-1.00
ubiquitin conjugation factor E4 A isoform 2	123.0	0.0	0.0	5.9	0.1	N/A	1.00
peroxisomal NADH pyrophosphatase NUDT12	52.0	0.0	2.5	0.0	0.1	N/A	-1.00
multifunctional protein ADE2-like	47.0	0.0	2.2	0.0	0.1	N/A	-1.00
peptidyl-prolyl cis-trans isomerase FKBP9	48.0	0.0	0.7	1.5	0.1	N/A	0.34
4-trimethylaminobutyraldehyde dehydrogenase, partial	56.0	0.0	2.6	0.0	0.1	N/A	-1.00
endoplasmic reticulum resident protein 44	39.0	0.0	0.0	1.7	0.1	N/A	1.00
anoctamin-10 isoform 1	76.0	0.0	0.0	3.3	0.1	N/A	1.00
arylsulfatase D	77.0	0.0	0.7	2.5	0.1	N/A	0.54
uncharacterized family 31 glucosidase KIAA1161	81.0	0.0	1.5	1.7	0.1	N/A	0.05
solute carrier family 25 member 46	37.0	0.0	1.5	0.0	0.1	N/A	-1.00
lon protease homolog, mitochondrial isoform 1	107.0	0.0	4.2	0.0	0.1	N/A	-1.00
probable cation-transporting ATPase 13A1	133.0	0.0	1.6	3.4	0.1	N/A	0.36
microsomal triglyceride transfer protein large subunit	100.0	0.0	0.0	3.4	0.1	N/A	1.00
desmocollin-2 isoform 3	101.0	0.0	0.8	2.6	0.1	N/A	0.51
peroxisome biogenesis factor 1 isoform 1	141.0	0.0	4.7	0.0	0.1	N/A	-1.00
long-chain-fatty-acid--CoA ligase 4 isoform 4	74.0	0.0	0.0	2.4	0.1	N/A	1.00
uncharacterized protein C3orf21	54.0	0.0	0.0	1.7	0.1	N/A	1.00

	plastin-3	68.0	0.0	2.1	0.0	0.1	N/A	-1.00
	fibrinogen beta chain isoform 2	55.0	0.0	0.0	1.7	0.1	N/A	1.00
	alpha-1-syntrophin isoform 1	55.0	0.0	0.0	1.7	0.1	N/A	1.00
	probable carboxypeptidase PM20D1	55.0	0.0	0.0	1.7	0.1	N/A	1.00
	abhydrolase domain-containing protein 2 isoform 3	48.0	0.0	1.5	0.0	0.1	N/A	-1.00
	cytochrome P450 2J2	58.0	0.0	0.0	1.7	0.1	N/A	1.00
	GRINL1A complex locus protein 1 isoform 2	64.0	0.0	0.0	1.9	0.1	N/A	1.00
	aladin isoform 2	59.0	0.0	0.0	1.7	0.1	N/A	1.00
	hepatocyte nuclear factor 3-alpha	77.0	0.0	0.0	2.1	0.1	N/A	1.00
	tetratricopeptide repeat protein 13 isoform 3	91.0	0.0	0.8	1.7	0.1	N/A	0.37
	cocaine esterase isoform 4	63.0	0.0	0.0	1.7	0.1	N/A	1.00
	transmembrane protein 201	70.0	0.0	0.0	1.7	0.1	N/A	1.00
	protein disulfide-isomerase A5	60.0	0.0	1.5	0.0	0.1	N/A	-1.00
	steryl-sulfatase	68.0	0.0	0.0	1.7	0.1	N/A	1.00
	E3 ubiquitin-protein ligase synoviolin	67.0	0.0	0.0	1.6	0.1	N/A	1.00
	transportin-1	106.0	0.0	0.8	1.7	0.1	N/A	0.37
	ATP-binding cassette sub-family A member 6 isoform 2	184.0	0.0	2.8	1.4	0.1	N/A	-0.33
	Ser/Thr-protein phosphatase 2A 65 kDa regulatory subunit A alpha isoform	65.0	0.0	1.5	0.0	0.1	N/A	-1.00
	cleft lip and palate transmembrane protein 1 isoform 1	76.0	0.0	0.0	1.7	0.1	N/A	1.00
	atrial natriuretic peptide-converting enzyme	114.0	0.0	0.0	2.4	0.1	N/A	1.00
	matrix metalloproteinase-9 precursor	78.0	0.0	1.5	0.0	0.1	N/A	-1.00
	vitamin K-dependent gamma-carboxylase	87.0	0.0	0.0	1.7	0.1	N/A	1.00
	neuroblastoma-amplified sequence	268.0	0.0	0.7	4.0	0.0	N/A	0.71
	fibronectin	271.0	0.0	2.4	1.5	0.0	N/A	-0.24
	nck-associated protein 1	126.0	0.0	1.6	0.0	0.0	N/A	-1.00
	macrophage mannose receptor 1	170.0	0.0	1.3	0.8	0.0	N/A	-0.26
	sodium channel protein type 5 subunit alpha	227.0	0.0	0.7	1.5	0.0	N/A	0.37
	myosin-10 isoform 4	229.0	0.0	2.0	0.0	0.0	N/A	-1.00
	voltage-dependent L-type calcium channel subunit alpha-1C	251.0	0.0	2.0	0.0	0.0	N/A	-1.00
	cation-independent mannose-6-phosphate receptor precursor	275.0	0.0	0.0	1.5	0.0	N/A	1.00
